# Metabolites of Key Flavor Compound 2,3,5-Trimethylpyrazine in Human Urine

**DOI:** 10.1021/acs.jafc.2c06418

**Published:** 2022-11-18

**Authors:** Dong Liang, Sebastian Dirndorfer, Veronika Somoza, Dietmar Krautwurst, Roman Lang, Thomas Hofmann

**Affiliations:** † Leibniz Institute for Food Systems Biology at the Technical University Munich, Lise-Meitner-Str. 34, 85354 Freising, Germany; ‡ Chair for Food Chemistry and Molecular Sensory Science, Technical University Munich, Lise-Meitner-Str. 34, 85354 Freising, Germany

**Keywords:** coffee, trimethylpyrazine, trimethylpyrazine metabolite synthesis, quantitative stable-isotope-dilution-UHPLC−MS/MS analysis, human urine

## Abstract

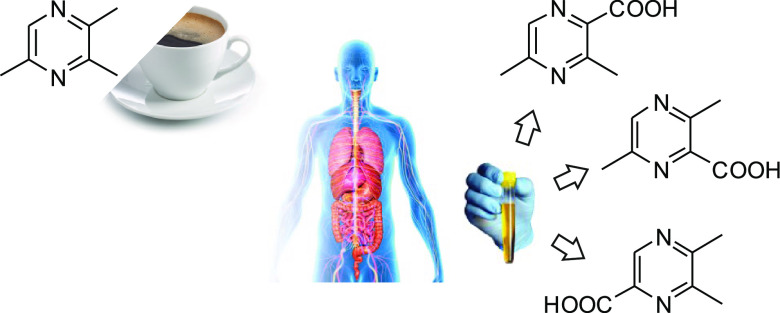

Pyrazines are among the most important compound class conveying the odor impressions “roasty”, “nutty”, and “earthy”. They are formed by the Maillard reaction and occur ubiquitously in heated foods. The excretion of metabolites of the key flavor odorant 2,3,5-trimethylpyrazine, abundant in the volatile fraction of roasted coffee, was investigated. Based on literature suggestions, putative phase 1 and phase 2 metabolites were synthesized, characterized by nuclear magnetic resonance and mass spectroscopy data and used as standards for targeted, quantitative analysis of coffee drinkers’ urine using stable-isotope-dilution-ultrahigh-performance liquid chromatography tandem mass spectroscopy (SIDA-UHPLC–MS/MS). The analysis of spot urine samples from a coffee intervention study revealed 3,6-dimethylpyrazine-2-carboxylic acid, 3,5-dimethylpyrazine-2-carboxylic acid, and 5,6-dimethylpyrazine-2-carboxylic acid were quantitatively dominating metabolites. Only negligible traces of pyrazinemethanols (3,6-dimethyl-2-pyrazinemethanol and 3,5,6-trimethylpyrazine-2-ol), glucuronides ((3,6-dimethylpyrazine-2-yl-)methyl-O-β-D-glucuronide and (3,5-dimethylpyrazine-2-yl-)methyl-O-β-D-glucuronide), and sulfates ((3,6-dimethylpyrazine-2-yl-)methyl-sulfate and (3,5-dimethylpyrazine-2-yl-)methyl-sulfate) were detected.

## 1 Introduction

Pyrazines, a compound class of aromatic heterocycles with nitrogen in the 1- and 4 position, are considered one of the most important compound class conveying earthy, nutty, and roasty odors.^1,2^ Pyrazines are common in heated foods, like roasted meat, bread, cocoa, or coffee,^3,4^ as they are naturally formed during nonenzymatic browning (Maillard) reaction from amino acids and reducing sugars upon heating. This reaction takes place in both aqueous and dry systems at elevated temperature,^5^ even under physiological conditions.^6^ Pyrazines in foods can also be products of microbial origin, for example, during fermentation processes.^4^ Pyrazines have low odor thresholds and are among the compounds used in the food industry as flavor ingredients with GRAS status (generally recognized as safe) confirmed by the FEMA (Flavor Extract Manufacturing Association).^2^ Despite their broad abundance in foods,^3,4^ literature on uptake, metabolism, and excretion of dietary alkyl-substituted pyrazines is limited and mainly comprises data from animal experiments.^2^ Reported pathways primarily involve cytochrome P-450-catalyzed oxidation of ring-substituted alkyl groups to form primary alcohols and subsequently carboxylic acids, while ring-hydroxylation apparently only occurs for selected pyrazines. It has been suggested that phase 1 metabolites formed by oxidation and hydroxylation may further be excreted in the urine after conjugation with glucuronides and sulfates because these are among the most important metabolic detoxication routes.^7^ However, sound literature in humans is lacking.^2,4,8^

Besides socializing aspects, the roasty aroma and the bitter taste of coffee brew is one of the key drivers for its consumption. Pyrazines substantially contribute to the aroma of coffee,^9−11^ and total pyrazine concentrations of 82.1–211.6 mg/kg have been reported in commercial roast coffee powder. The derivative 2-methylpyrazine was of highest abundance followed by 2,5-dimethylpyrazine and 2,6-dimethylpyrazine.^12^ Pyrazines are effectively extracted into the brew during coffee making, with extraction rates reaching 82%.^13^ A recent human coffee intervention study reported that the pyrazines 2-methylpyrazine, 2,5-dimethylpyrazine, and 2,6-dimethylpyrazine in coffee were metabolized into the corresponding 2-carboxylic acid derivatives and excreted via the urine.^14^ In coffee, the roasty smelling 2,3,5-trimethylpyrazine (TMP, **1**) occurs in concentrations between 1 and 6.7 mg/kg and has an odor threshold of ∼50 ng/L air.^12,15^

**Figure 1:**
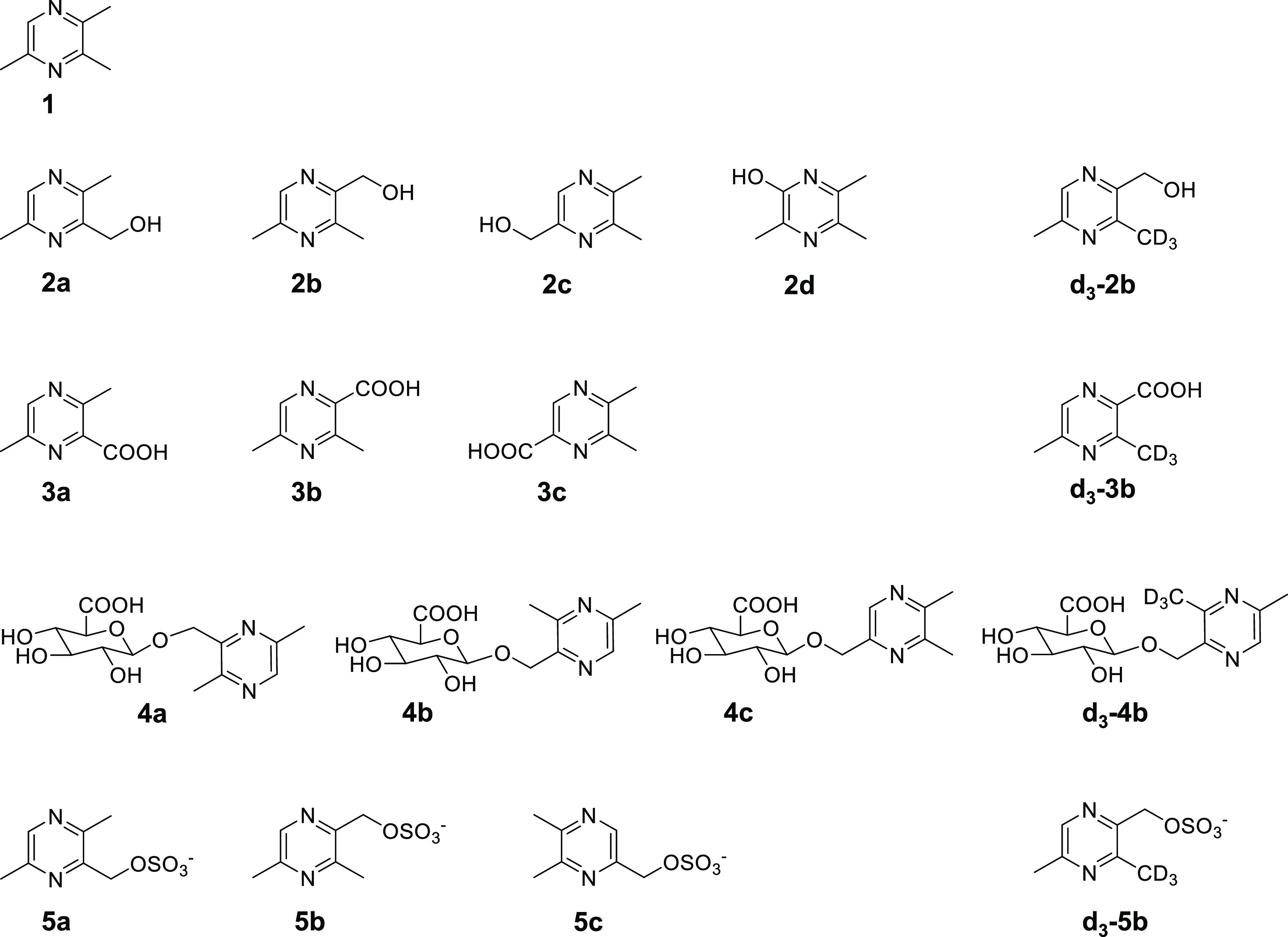
Chemical structures of the target compound TMP (**1**) and the putative metabolites 3,6-dimethyl-2-pyrazinemethanol (**2a**), 3,5-dimethyl-2-pyrazinemethanol (**2b**), 5,6-dimethyl-2-pyrazinemethanol (**2c**), 3,5,6-trimethylpyrazine-2-ol (**2d**), 3,6-dimethylpyrazine-2-carboxylic acid (**3a**), 3,5-dimethylpyrazine-2-carboxylic acid (**3b**), 5,6-dimethylpyrazine-2-carboxylic acid (**3c**), (3,6-dimethylpyrazine-2-yl-)methyl-O-β-D-glucuronide (**4a**), (3,5-dimethylpyrazine-2-yl-)methyl-O-β-D-glucuronide (**4b**), (5,6-dimethylpyrazine-2-yl-)methyl-O-β-D-glucuronide (**4c**), (3,6-dimethylpyrazine-2-yl-)methyl-sulfate (**5a**), (3,5-dimethylpyrazine-2-yl-)methyl-sulfate (**5b**), (5,6-dimethylpyrazine-2-yl-)methyl-sulfate (**5c**), and the isotope labeled internal standards 3-d_3_,5-dimethyl-2-pyrazinemethanol (**d_3_-2b**), 3-d_3_,5-dimethylpyrazine-2-carboxylic acid (**d_3_-3b**), (3-d_3_,5-dimethylpyrazine-2-yl-)methyl-O-β-D-glucuronide (**d_3_-4b**), and (3-d_3_,5-dimethylpyrazine-2-yl-)methyl-sulfate (**d_3_-5b**).

The focus of the present study was the key flavor compound TMP. Based on the available information, it was the aim to synthesize putative phase 1 and 2 metabolites of TMP (Figure 1) in order to quantitate excreted metabolites of TMP in human urine.

## 2 Materials and Methods

### 2.1 Chemicals

L-alaninamide hydrochloride, 2,3-pentanedione, 2,3-diaminopropionic acid, trimethylpyrazine, sodium methoxide solution (25%), acetobromo-α-D-glucuronic acid methyl ester, 2,6-di-*tert*-butyl-4-methylpyridine, sulfur trioxide pyridine complex, 2-chloro-3,5-dimethylpyrazine, methyl-d_3_-magnesium iodide, iron(III) acetylacetonate, and 3-chloro-2,5-dimethylpyrazine were purchased from Sigma Aldrich (Sigma Aldrich, Taufkirchen, Germany). Diacetyl and acetic anhydride were purchased from Merck Schuchardt OHG (Hohenbrunn, Germany). Hydrogen peroxide (H_2_O_2_) and acetic acid (glacial) were purchased from Merck KGaA (Darmstadt, Germany). Sodium borohydride (NaBH_4_), potassium permanganate (KMnO_4_), and silver trifluoromethanesulfonate were purchased from Fluka (Steinheim, Germany). Water for HPLC separation was purified by means of a Milli-Q Advantage A10 water system (Millipore, Molsheim, France). Artificial urine (AU) for analytical purposes was prepared according to Sarigul et al.^16^ The compounds 3,6-dimethyl-2-pyrazinemethanol (**2a**), 3,5-dimethyl-2-pyrazinemethanol (**2b**), 5,6-dimethyl-2-pyrazinemethanol (**2c**), 3,5,6-trimethylpyrazine-2-ol (**2d**), 3,6-dimethylpyrazine-2-carboxylic acid (**3a**), 3,5-dimethylpyrazine-2-carboxylic acid (**3b**), 5,6-dimethylpyrazine-2-carboxylic acid (**3c**), (3,6-dimethylpyrazine-2-yl-)methyl-O-β-D-glucuronide (**4a**), (3,5-dimethylpyrazine-2-yl-)methyl-O-β-D-glucuronide (**4b**), (5,6-dimethylpyrazine-2-yl-)methyl-O-β-D-glucuronide (**4c**), (3,6-dimethylpyrazine-2-yl-)methyl-sulfate (**5a**), (3,5-dimethylpyrazine-2-yl-)methyl-sulfate (**5b**), (5,6-dimethylpyrazine-2-yl-)methyl-sulfate (**5c**), and the isotope labeled internal standards 3-d_3_,5-dimethyl-2-pyrazinemethanol (**d_3_-2b**), 3-d_3_,5-dimethylpyrazine-2-carboxylic acid (**d_3_-3b**), (3-d_3_,5-dimethylpyrazine-2-yl-)methyl-O-β-D-glucuronide (**d_3_-4b**), and (3-d_3_,5-dimethylpyrazine-2-yl-)methyl-sulfate (**d_3_-5b**) were synthesized (refer to next chapter 2.1).

### 2.2 Synthesis of Compounds

#### 2.2.1 3,6-Dimethyl-2-pyrazinemethanol (**2a**) and 3,5-Dimethyl-2-pyrazinemethanol (**2b**)

2,3,5-Trimethylpyrazine (12.2 g, 100 mmol) was slowly added to glacial acetic acid (30 mL) at 0 °C.^17^ The mixture was allowed to reach room temperature and was then mixed with H_2_O_2_ (30%, 30 mL). Upon heating to 58 °C, further H_2_O_2_ (30%, 30 mL) was added and stirred for 16 h. Water (80 mL) was added and the mixture evaporated to 10% of its original volume, and this procedure was repeated three times. The pH was adjusted to 9 with saturated K_2_CO_3_ solution, and the solution was extracted with DCM four times. The combined organic layers were washed with saturated brine, dried over anhydrous sodium sulfate, and concentrated to give the mixed *N*-oxides (13.21 g). The mixture of trimethylpyrazine *N*-oxides (12.48 g) was mixed with an excess of acetic anhydride (70 mL) and heated (100 °C, 20 h. After reaching room temperature, the solution was poured on ice and adjusted to pH 9 with solid K_2_CO_3_. The mixture was extracted three times with DCM. The organic layers were combined and dried over anhydrous sodium sulfate to afford the crude acetates (14.20 g). The crude acetates (6.42 g) were incubated with NaOH (5 M, 50 mL) at room temperature overnight to achieve hydrolysis. The resulting solution was extracted three times with DCM and after evaporation gave a brown oil (4.73 g). The oil was chromatographed on silica gel with ethyl acetate to afford the crude mixture (2.12 g), which was then taken up in 20 mL of H_2_O/acetonitrile (96:4) and separated on a preparative RP18-HPLC. Isocratic elution with 0.1% formic acid/acetonitrile (96:4) gave two major peaks. The collected aqueous fractions were extracted with DCM four times, dried over anhydrous sodium sulfate, and evaporated to afford 2-pyrazinemethanol-3,6-dimethyl (631 mg, yield = 11%) and 2-pyrazinemethanol-3,5-dimethyl (281 mg, yield = 5%).

#### 2.2.2 3,6-Dimethyl-2-pyrazinemethanol (**2a**)

^1^H NMR (400 MHz, CDCl_3_): δ ppm 8.08 (s, 1 H), 4.60 (s, 2 H), 2.40 (s, 3 H), 2.35 (s, 3 H). ^13^C NMR (100 MHz, CDCl_3_, HMBC): δ ppm 151.8 (C-6), 150.7 (C-2), 149.5 (C-3), 140.1 (C-5), 62.1 (C-7), 21.5 (C-9), 20.6 (C-8). LC-TOF-MS: *m/z* = 139.08688, [M + H]^+^, calcd for C_7_H_11_N_2_O, 139.0871. MS/MS (ACN/H_2_O 1/1, ESI^+^) 139.1 (100), 121.0 (26), 109.0 (4), 80.0 (4), 53 .0 (4), 42.2 (2), 39.0 (4).

#### 2.2.3 3,5-Dimethyl-2-pyrazinemethanol (**2b**)

^1^H NMR (400 MHz, CDCl_3_): δ ppm 8.18 (s, 1 H), 4.64 (s, 2 H), 2.45 (s, 3 H), 2.37 (s, 3 H). ^13^C NMR (100 MHz, CDCl_3_, HMBC): δ ppm 151.2 (C-3), 149.8 (C-5), 148.3 (C-2), 142.3 (C-6), 61.8 (C-7), 21.4 (C-9), 20.0 (C-8). LC-TOF-MS: *m/z* = 139.0868, [M + H]^+^, calcd for C_7_H_11_N_2_O, 139.0869. MS/MS (ACN/H_2_O 1/1, ESI^+^) 139.1 (100), 121.0 (20), 109.0 (4), 80.0 (3), 53.0 (4), 42.2 (2), 39.0 (4).

#### 2.2.4 3,5,6-Trimethylpyrazin-2-ol (**2d**)

L-alaninamide hydrochloride (1.24 g, 10 mmol) was dissolved in an NaOH solution (6 mL, 5 M) mixed with 2,3-butanedione (0.86 g, 10 mmol) in water (4 mL) and stirred at room temperature for 24 h.^18,19^ The pH was adjusted to 6–7 with concentrated HCl and the mixture was extracted with DCM three times. The combined organic layers were evaporated and gave a brown powder. Recrystallization in DCM/pentane (1:6) gave the title compound (110 mg, yield = 8%). ^1^H NMR (400 MHz, CDCl_3_): δ ppm 2.39 (s, 3 H), 2.27 (s, 3 H), 2.23 (s, 3 H). ^13^C NMR (100 MHz, CDCl_3_): δ ppm 158.8153.2, 131.6, 130.3, 20.3, 19.3, 16.7. LC-TOF-MS: *m/z* = 139.0898, [M + H]^+^, calcd for C_7_H_11_N_2_O, 139.0871. MS/MS (ACN/H_2_O 1/1, ESI^+^) 139.1 (100), 111.0 (3), 70.0 (2), 42.0 (5).

#### 2.2.5 5,6-Dimethylpyrazine-2-carboxylic acid (**3c**)

2,3-Diaminopropionic acid hydrochloride (5 g, 35.7 mmol) was dissolved in anhydrous methanol (320 mL) and solid NaOH (5.7 g, 143 mmol) was added.^20^ Then, diacetyl (3.10 g, 35.7 mmol) was added and a stream of oxygen was bubbled through the solution for 4 h under stirring. Stirring further continued for 20 h at room temperature. The solvent was evaporated, the residue taken up with water (50 mL), and again evaporated to dryness. The residue was dissolved in water (50 mL), adjusted to pH 2.0 with concentrated hydrochloric acid, and extracted four times with DCM. The combined organic layers were dried over anhydrous sodium sulfate and filtered. After removal of the solvent, the yellow solid was further crystallized in hot methanol/ethyl acetate (1:1) to afford the title compound as light-yellow crystals (1.53 g, yield 28%).^1^H NMR (600 MHz, CDCl_3_): δ ppm 9.14 (s, 1 H), 2.68 (s, 3 H), δ 2.64 (s, 3 H). ^13^C NMR (150 MHz, CDCl_3_) δ ppm 164.6, 158.9, 152.4, 143.0, 138.6, 23.2, 22.6. LC-TOF-MS: *m/z* = 153.0668, [M + H]^+^, calcd for C_7_H_9_N_2_O_2_, 153.0664. MS/MS (ACN/H_2_O 1/1, ESI^+^) 153.1 (100), 135.0 (15), 109.2 (6), 107.0 (35), 66.0 (22), 53.0 (6), 42.0 (2), 39.0 (2).

#### 2.2.6 5,6-Dimethyl-2-pyrazinemethanol (**2c**)

5,6-Dimethyl-2-carboxylic acid (**3c**, 1.28 g, 7.7 mmol) was dissolved in anhydrous methanol (15 mL), mixed with a methanolic solution of sodium methoxide (83 μL, 25%, 0.38 mmol) and NaBH_4_ (760 mg, 20 mmol), and stirred overnight.^21^ The reaction was terminated by adding excess amount of methanol and then evaporated under reduced pressure. The obtained oil was washed with water and extracted three times with DCM. The organic layers were combined and evaporated. The crude residue was purified with silica gel chromatography (ethyl acetate/hexane, 1:1) to afford the title product as a crystalline solid (537 mg, yield = 50%). ^1^H NMR (500 MHz, CDCl_3_): δ ppm 8.30 (s, 1 H), 4.74 (s, 2 H), 2.45 (s, 3 H), 2.53 (s, 6 H). ^13^C NMR (125 MHz, CDCl_3_): δ ppm 152.1, 151.9, 151.5, 139.7, 63.2, 22.6, 22.4. LC-TOF-MS: *m/z* = 139.0881, [M + H]^+^, calcd for C_7_H_11_N_2_O, 139.0869. MS/MS (ACN/H_2_O 1/1, ESI^+^) 139.1 (100), 121.0 (6), 109.0 (11), 80.0 (4), 42.0 (2), 39.0 (4).

#### 2.2.7 3,6-Dimethylpyrazine-2-carboxylic acid (**3a**)

2-Pyrazinemethanol-3,6-dimethyl (80 mg, 0.58 mmol) was dissolved in distilled water (2 mL) and slowly added to KMnO_4_ (128 mg, 0.81 mmol, in 2 mL H_2_O) within 30 min with stirring. Stirring continued for another 30 min, and then, the mixture was centrifuged at 10,000 *g* for 10 min at room temperature to precipitate MnO_2_. The supernatant was collected and acidified with 2 drops of concentrated hydrochloric acid and subsequently extracted with DCM four times. The organic layers were combined, dried over anhydrous sodium sulfate, and evaporated. The obtained solid (58.4 mg) was recrystallized from hot ethyl acetate/methanol (1:1) to give the title compound (23 mg, yield = 26%). ^1^H NMR (500 MHz, CDCl_3_): δ ppm 8.30 (s, 1 H), 2.98 (s, 3 H), δ 2.67 (s, 3 H). ^13^C NMR (125 MHz, CDCl_3_) δ = 164.2, 159.0, 157.1, 140.3, 136.7, 24.1, 22.7. LC-TOF-MS: *m/z* = 153.0667, [M + H]^+^ (calcd for C_7_H_9_N_2_O_2_, 153.0664); *m/z* = 151.0503, [M – H]^−^, calculated for C_7_H_7_N_2_O_2_, 151.0507. MS/MS (ACN/H_2_O 1/1, ESI^+^) 153.1 (100), 135.0 (12), 109.0 (15), 107.0 (15), 66.0 (6), 42.0 (7).

#### 2.2.8 3,5-Dimethylpyrazine-2-carboxylic acid (**3b**)

This compound was prepared from 2-pyrazinemethanol-3,5-dimethyl using the same method as described for **3a**. Yield = 33%. ^1^H NMR (500 MHz, CDCl_3_): δ ppm 8.64 (s, 1 H), 2.97 (s, 3 H), 2.62 (s, 3 H). ^13^C NMR (125 MHz, CDCl_3_): δ ppm 164.2, 154.7, 150.2, 148.8, 138.0, 23.6, 21.4. LC-TOF-MS: *m/z* = 153.0667, [M + H]^+^, calcd for C_7_H_9_N_2_O_2_, 153.0664. MS/MS (ACN/H_2_O 1/1, ESI^+^) 153.1 (100), 135.0 (12), 109.0 (7), 107.0 (28), 80.0 (10), 66.0 (3), 42.0 (5).

### 2.3 Synthesis of Glucuronides

2,6-di-*tert*-Butyl-4-methylpyridine (205 mg, 1 mmol), silver trifluoromethanesulfonate (640 mg, 2.5 mmol), and molecular sieves (4 Å, 2 g) were suspended in dry 1,2-dichloroethane (15 mL) under a nitrogen atmosphere.^23^ The mixture was cooled to −20 °C, and dimethyl pyrazinemethanol (138 mg, 1 mmol) and acetobromo-α-D-glucuronic acid methyl ester (794 mg, 2 mmol) were added. After stirring for 20 min, the reaction was allowed to reach room temperature, and another portion of 2,6-di-*tert*-butyl-4-methylpyridine (205 mg, 1 mmol) was added and stirred overnight. The solid was filtered off and the residue obtained after evaporation was chromatographed on silica gel (ethyl acetate/hexane 3:1) to afford the crude methyl esters, which were hydrolyzed with NaOH (2 M, 10 mL, MeOH/H_2_O 1:1) overnight. The hydrolysate was neutralized with concentrated hydrochloric acid, the solvent was removed, and the residue was suspended in H_2_O. The mixture was separated by preparative RP18-HPLC (isocratic 7% ACN), the individual compounds collected, and freeze-dried to give the glucuronides.

#### 2.3.1 (3,6-Dimethylpyrazine-2-yl-)methyl-O-β-D-glucuronide (**4a**)

(31.4 mg, yield = 10%). ^1^H NMR (400 MHz, D_2_O): δ ppm 8.36 (s, 1 H), 5.05–4.96 (m, 2 H), 4.59 (d, 1 H, *J* = 7.9 Hz), 3.87 (d, 2 H, *J* = 9.0 Hz), 3.57–3.49 (m, 2 H), 3.38 (t, 1 H, *J* = 8.3 Hz), 2.60 (s, 3 H), 2.54 (s, 3 H). ^13^C NMR (100 MHz, D_2_O): δ ppm 173.5, 153.2, 152.9, 147.7, 141.5, 102.5, 75.9, 75.5, 73.2, 71.9, 70.3, 20.1, 19.9. LC-TOF-MS: *m/z* = 315.1186, [M + H]^+^, calcd for C_13_H_19_N_2_O_7_, 315.1192). MS/MS (ACN/H_2_O 1/1, ESI^+^) 315.1 (100), 139.0 (62), 121.0 (98), 80.0 (17).

#### 2.3.2 (3,5-Dimethylpyrazine-2-yl-)methyl-O-β-D-glucuronide (**4b**)

(23 mg, yield = 7.3%). ^1^H NMR (400 MHz, D_2_O): δ ppm 8.39 (s, 1 H), 5.00 (s, 2 H), 4.63 (d, 1 H, *J* = 7.89 Hz), 3.92(d, 2 H, 9.15 Hz), 3.58–3.50 (m, 2 H), 3.39 (t, 1 H, *J* = 8.5 Hz), 2.60 (s, 3 H), 2.54 (s, 3 H). ^13^C NMR (100 MHz, D_2_O): 172.6, 152.2, 150.9, 149.7140.8, 103.0, 75.7, 75.0, 73.1, 71.7, 70.0, 20.0, 18.8. LC-TOF-MS: *m/z* = 315.1194, [M + H]^+^, calcd for C_13_H_19_N_2_O_7_, 315.1192. MS/MS (ACN/H_2_O 1/1, ESI^+^) 315.1 (100), 139.2 (60), 121.2 (58).

#### 2.3.3 (5,6-Dimethylpyrazine-2-yl-)methyl-O-β-D-glucuronide (**4c**)

(37 mg, yield = 12%). ^1^H NMR (400 MHz, D_2_O): δ ppm 8.42 (s, 1 H), 4.97–4.87 (m, 2 H), 4.65 (d, 1 H, *J* = 7.88 Hz), 3.93 (d, 1 H, *J* = 9.2 Hz), 3.55 (m, 2 H), 3.40 (t, 1 H, *J* = 8.4 Hz), 2.56 (s, 6 H). ^13^C NMR (100 MHz, D_2_O): 172.6, 153.8, 152.2, 148.5, 138.4, 102.1, 75.2, 74.6, 72.7, 71.2, 69.6, 20.5, 20.0. LC-TOF-MS: *m/z* = 315.1183, [M + H]^+^, calcd for C_13_H_19_N_2_O_7_, 315.1192. MS/MS (ACN/H_2_O 1/1, ESI^+^) 315.1 (100), 139.2 (95), 121.2 (59), 109.0 (17), 80.0 (32).

### 2.4 Synthesis of Sulfates

The sulfur trioxide pyridine complex (32 mg, 0.2 mmol) and dimethyl pyrazinemethanol (27.6 mg, 0.2 mmol) were dissolved in dry acetonitrile (2 mL).^23^ The vessel was sealed and heated to 100 °C for 2 h. After cooling to room temperature, the solvent was removed under reduced pressure, the remaining solid was dissolved in water (5 mL), purified with preparative RP-HPLC, and freeze-dried to afford the final product as white powder.

#### 2.4.1 (3,6-Dimethylpyrazine-2-yl-)methyl-sulfate (**5a**)

6.9 mg, yield = 16%. ^1^H NMR (400 MHz, D_2_O): δ ppm 8.73 (s, 1 H), 5.31 (s, 2 H), 2.76 (s, 3 H), 2.70 (s, 3 H). ^13^C NMR (100 MHz, D_2_O): δ ppm 150.6, 150.3, 149.4, 144.3, 68.1, 18.5, 18.3. LC-TOF-MS: *m/z* = 217.0285, [M – H]^−^, calcd for C_13_H_17_N_2_O_7_, 217.0283. MS/MS (ACN/H_2_O 1/1, ESI^–^) 216.9 (100), 97.0 (52), 80.0 (41).

#### 2.4.2 (3,5-Dimethylpyrazine-2-yl-)methyl-sulfate (**5b**)

5.6 mg, yield = 13.4%. ^1^H NMR (600 MHz, D_2_O): δ ppm 8.53 (s, 1 H), 5.29 (s, 2 H), 2.72 (s, 3 H), 2.65 (s, 3 H). ^13^C NMR (150 MHz, D_2_O): δ ppm 153.3, 150.1, 149.0, 140.1, 68.1, 20.2, 18.5. LC-TOF-MS: *m/z* = 217.0284, [M – H]^−^, calcd for C_13_H_17_N_2_O_7_, 217.0283. MS/MS (ACN/H_2_O 1/1, ESI^–^) 216.9 (100), 96.8 (38), 80.0 (75).

#### 2.4.3 (5,6-Dimethylpyrazine-2-yl-)methyl-sulfate (**5c**)

(13.1 mg, yield = 30%). ^1^H NMR (500 MHz, D_2_O): δ ppm 8.52 (s, 1 H), 5.21 (s, 2 H), 2.67 (s, 3 H), 2.66 (s, 3 H). ^13^C NMR (125 MHz, D_2_O): δ ppm 157.5, 150.9, 150.3, 134.4, 67.8, 21.3, 19.3. LC-TOF-MS: *m/z* = 217.0287, [M – H]^−^, calcd for C_13_H_17_N_2_O_7_, 217.0283. MS/MS (ACN/H_2_O 1/1, ESI^–^) 216.8 (100), 96.0 (82), 80.0 (100).

### 2.5 Synthesis of Stable Isotope Labeled Standards

#### 2.5.1 2,3-d_3_,5-Trimethylpyrazine (**1b**)

3-Chloro-2,5-dimethylpyrazine (20.16 g, 142 mmol) and iron(III) acetylacetonate (3.18 g, 9 mmol) were dissolved in dry diethyl ether (1 L).^12^ The solution was cooled to 0 °C and a solution of methyl-d_3_-magnesium iodide (1 M in ether, 200 mL, 200 mmol) was added dropwise. The mixture was stirred overnight and carefully quenched with diluted hydrochloric acid (1 M, 100 mL). The organic layer was dried with anhydrous sodium sulfate and concentrated. The crude product was purified on silica gel (pentane/ether 7:3) to afford the title compound (3.86 g, yield = 22%). ^1^H NMR (CD_3_Cl, 500 MHz): δ ppm 8.16 (s, 1 H), 2.50 (s, 3 H), 2.48 (s, 3 H). ^13^C NMR (CD_3_Cl, 125 MHz): δ ppm 151.3, 150.7, 144.2, 141.5, 21.8, 21.7. LC-TOF-MS: *m/z* = 126.1131, [M + H]^+^, calcd for C_7_H_8_D_3_N_2_, 126.1111.

Starting from **1b**, deuterium-substituted metabolites **d_3_-2b**, **d_3_-3b**, **d_3_-4b**, and **d_3_-5b** were synthesized similarly to their hydrogen counterparts describe above.

#### 2.5.2 3-d_3_,5-Dimethyl-2-pyrazinemethanol (**d_3_-2b**)

^1^H NMR (400 MHz, CDCl_3_): δ ppm 8.24 (s, 1 H), 4.72 (s, 2 H), 2.54 (s, 3 H). ^13^C NMR (100 MHz, CDCl_3_, HMBC): δ ppm 152.0 (C-5), 150.5 (C-3), 149.4 (C-2), 140.2 (C-6), 61.9 (C-7), 21.8 (C-9). LC-TOF-MS: *m/z* = 142.1057, [M + H]^+^, calcd for C_7_H_8_D_3_N_2_O, 142.1059. MS/MS (ACN/H_2_O 1/1, ESI^+^) 142.1 (100), 124.0 (20), 112.0 (2), 80.0 (2), 39.0 (4).

#### 2.5.3 3-d_3_,5-Dimethylpyrazine-2-carboxylic acid (**d_3_-3b**)

^1^H NMR (600 MHz, CD_3_OD): δ ppm 8.41 (s, 1 H), 2.59 (s, 3 H). ^13^C NMR (150 MHz, CD_3_OD): δ ppm 168.0, 157.7, 155.5, 142.1, 141.1, 21.5. LC-TOF-MS: *m/z* = 156.0856, [M + H]^+^, calcd for C_7_H_6_D_3_N_2_O_2_,156.0852. MS/MS (ACN/H_2_O 1/1, ESI^+^) 156.1 (100), 138.0 (20), 112.0 (8), 110.0 (30), 66.0 (8), 45.0 (12), 39.0 (5).

#### 2.5.4 (3-d_3_,5-Dimethylpyrazine-2-yl-)methyl-O-β-D-glucuronide (**d_3_-4b**)

^1^H NMR (600 MHz, CD_3_CN): δ ppm 8.23 (s, 1 H), 4.95 (d, 1 H, *J* = 12.5 Hz), 4.77 (d, 1 H, *J* = 12.5 Hz), 4.43 (d, 1 H, *J* = 7.83 Hz), 3.79 (d, 1 H, *J* = 9.79 Hz), 3.49 (t, 1 H, *J* = 9.37 Hz), 3.33 (t, 1 H, *J* = 9.06 Hz), 3.21 (dd, 1 H, *J*_1_ = 7.90 Hz, *J*_2_ = 7.94 Hz), 2.46 (s, 3 H). ^13^C NMR (150 MHz, CD_3_CN): δ ppm 170.5, 153.5, 153.4, 148.0, 141.2, 103.7, 76.9, 75.3, 74.1, 72.3, 70.7, 21.3. LC-TOF-MS: *m/z* = 318.1378, [M + H]^+^, calcd for C_13_H_16_D_3_N_2_O_7_, 318.1380. MS/MS (ACN/H_2_O 1/1, ESI^+^) 318.1 (100), 142.2 (72), 124.0 (87).

#### 2.5.5 (3-d_3_,5-Dimethylpyrazine-2-yl-)methyl-sulfate (**d_3_-5b**)

^1^H NMR (600 MHz, D_2_O): δ ppm 8.72(s, 1 H), 5.31 (s, 2 H), 2.70 (s, 3 H). ^13^C NMR (150 MHz, D_2_O) δ ppm 150.6, 150.2, 149.2, 144.2, 68.0, 18.5. LC-TOF-MS: *m/z* = 220.0471, [M – H]^−^, calcd for C_7_H_6_D_3_N_2_O_4_S^–^, 220.0471. MS/MS (ACN/H_2_O 1/1, ESI^–^) 219.6 (100), 97.8 (48), 79.8 (37).

### 2.6 Coffee Intervention Study

**Figure 2:**
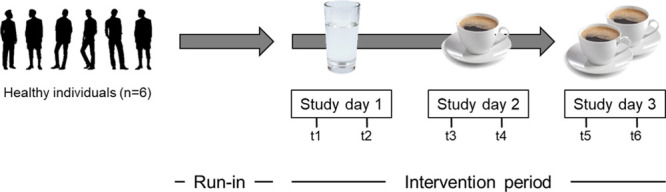
Design of the pilot coffee drinking study to investigate TMP metabolites in human urine.

Urine samples were from a recent pilot intervention study (cf. Figure 2) aimed at the identification of odor-active coffee-derived compounds in biosamples. It was approved by the ethical committee of the Faculty of Medicine at the Technical University Munich, ethical vote 357/20 S. The study was registered in “Deutsches Register Klinischer Studien”, DRKS00024380. The study protocol adhered to the Declaration of Helsinki for Human Intervention/clinical studies. Six healthy, nonsmoking participants with neither acute nor chronic diseases, no current medication, and no known orosensory disorders (m/w 3/3, age 25–43) participated in the study and gave written informed consent. In brief, the study participants went through a run-in phase (3 days) in which they were asked to refrain from the consumption of coffee, coffee-containing foods/sweets, caffeine-containing foods/drinks, and strongly flavored meals. The study phase followed immediately after and comprised three consecutive days. On each study day, the volunteers fasted for 10–15 h prior to admittance to the study center. A morning spot urine sample (sampling points t1, t3, t5) was collected. Then, either tap water (day one, control, 250 mL), a dose of coffee brew (day two, 250 mL), or two doses of coffee brew (day three, 500 mL) was consumed. Further spot urine samples were collected after 4 h (day one and two, time point t2 and t4) and 2 h (day three, t6; Figure 2). The administered coffee brew was freshly prepared as follows: whole roasted coffee beans (50 g, Tchibo Caffè Crema, 100% Arabica) were ground in a coffee mill (10 s) and passed through a sieve (2 mm). The ground material (48.75 g) was weighed into a French press, mixed with freshly boiled table water (Evian, 750 mL), and incubated (4 min). Finally, the press was passed through the suspension and the resulting brew (200 and 400 mL, respectively) was poured into cups. Aliquots were used for quantification of 2,3,5-trimethylpyrazine (**1**). The coffee brew contained 4.08 μM (±9.2%, *n* = 3) of compound **1** determined by stable isotope dilution analysis (GC–MS/MS) and an in-house protocol. One dose of coffee (200 mL, day 2) delivered 0.82 μmol and two doses of coffee (400 mL day 3) delivered 1.63 μmol of compound **1**.

### 2.7 Quantitative Analysis (UHPLC–MS/MS)

#### 2.7.1 Instrumentation

The UHPLC–MS/MS system consisted of a QTrap 6500+ MS/MS system (Sciex, Darmstadt, Germany) connected to an ExionLC UHPLC system (Sciex, Darmstadt, Germany). The MS instrument was operated in the ESI^+^ mode, applying a spray voltage of +5500 V and a source temperature of 600 °C. The nebulizer gas was set to 55 psi, the heating gas was set to 65 psi, and the curtain gas (nitrogen) was set to 35 psi. The MS/MS parameters, including the collision cell entrance potential (CEP), declustering potential (DP), collision energy (CE), and cell exit potential (CXP), were tuned for each individual compound and mass transition. For the chromatographic separation, a gradient elution at 50 °C on a biphenyl column (Kinetex Biphenyl, 150 × 2.1 mm, 1.7 μm, Phenomenex, Aschaffenburg, Germany) was performed using eluent A (0.1% formic acid, 1 mM NH_4_Ac in water), eluent B (0.1% formic acid, 1 mM NH_4_Ac in acetonitrile), and following gradient at a flow rate of 0.5 mL/min: 0 min 1% B, 2 min 5% B, 8 min 3% B, 9 min 50% B, 11 min 50% B, 11.5 min 1% B, and 15 min 1% B. The flow was directed to the MS from minute 1 to 8.

For analysis of sulfates, the MS operated in ESI^–^. Ion spray voltage was −4500 V, ion source temperature 550 °C, nebulizer gas was set to 55 psi, heating gas to 65 psi, and the curtain gas (nitrogen) was set to 35 psi. Chromatographic separation utilized gradient elution at 50 °C on a biphenyl column (Kinetex Biphenyl, 150 × 2.1 mm, 1.7 μm, Phenomenex, Aschaffenburg, Germany) with eluent A (0.1% formic acid, 1 mM NH_4_Ac in water) and eluent B (0.1% formic acid, 1 mM NH_4_Ac acetonitrile). At a flow rate of 0.6 mL/min, gradient elution was as follows: 0 min 1% B, 2 min 1% B, 4 min 10% B, 6 min 60% B, 6.3 min 60% B, 7 min 1% B, and 10 min 1% B. The injection volume was 5 μL. The flow was directed to the MS from minute 1 to 5.

#### 2.7.2 Stock Solutions and Calibration

Individual stock solutions of the synthesized reference compounds and the internal standards were prepared in deuterated methanol (d_4_-methanol) (range 8–34 mM determined by quantitative ^1^H NMR,^21^). Aliquots were subsequently combined and diluted in 50% aqueous ACN to prepare one separate mixture of reference compounds (mixed analyte solution) with a final concentration of 100 μM per compound and another separate mixture of internal standards (mixed IS solution) with a final concentration of 10 μM per compound. Artificial urine (AU^16^) was used as the matrix for calibration standards and quality controls. The mixed analyte stock was serially diluted with AU (1 + 1, v/v) to obtain dilutions from 1250 to 9 nM. Quality control samples (QCs) were prepared in AU in triplicates (*n* = 3) at 313 nM and analyzed in replicates (*n* = 6) to assess precision and accuracy. For the sulfates, calibration standards and QC samples were run in replicates (*n* = 5) (Table 1).

**Table 1:** Mass Transitions, Retention Times, Calibrated Range, Limit of Detection, and Lower Limit of Quantitation for the Analysis of Putative Metabolites of 2,3,5-Trimethylpyrazine

analyte		Q1 → Q3^a^	Rt. (min)	calibrated range (nM)	*R*^2^	LoD (nM)^b^	LloQ (nM)^c^	precision (%)^d^	accuracy (%)^d^	precision (%)^e^	accuracy (%)^e^
								calibration standards		quality controls	
3,6-dimethyl-2-pyrazinemethanol	**2a**	138.9 > 139.0, 121.1, 80.0*, 53.0	5.05 ± 0.03	9–625	0.9958	<9 (S/N 14)	9	3.9–11.5	91.7–112.6	3.6–7.9	94.5–101.0
3,5-dimethyl-2-pyrazinemethanol	**2b**	139.0 > 110.0*, 109.0, 80.0	5.49 ± 0.02	19–1250	0.9952	<19 (S/N 12)	19	3.5–21.3	95.7–109.3	5.1–6.2	98.2–107.1
5,6-dimethyl-2-pyrazinemethanol	**2c**	139.0 > 110.0, 109.0*, 80.0	5.90 ± 0.03	9–1250	0.9956	<9 (S/N 39)	9	2.7–12.1	94.6–109.2	2.2–3.5	100.1–105.8
3,5,6-trimethylpyrazine-2-ol	**2d**	139.0 > 111.0, 70.0*, 53.0	6.45 ± 0.03	19–1250	0.9896	<9 (S/N 29)	9	7.5–12.6	94.9–107.7	8.8–12.6	98.2–107.2
3,6-dimethylpyrazine-2-carboxylic acid	**3a**	153.0 > 109.0, 107.0*, 135.0, 80.0	4.35 ± 0.02	9–1250	0.9944	<9 (S/N 23)	9	3.4–19.2	94.5–106.2	6.1–8.4	99.5–104.2
3,5-dimethylpyrazine-2-carboxylic acid	**3b**	152.9 > 109.0, 135.0, 107*	4.95 ± 0.04	19–1250	0.9948	<19 (S/N 5)	39	5.3–16.9	94.9–104.0	6.9–13.2	96.9–101.3
5,6-dimethylpyrazine-2-carboxylic acid	**3c**	153.0 > 107.0, 135.0, 109.0, 66.0*	6.41 ± 0.03	9–1250	0.9940	<9 (S/N 38)	9	5.5–17.1	93.8–104.5	6.7–10.7	100.9–103.9
(3,6-dimethylpyrazine-2-yl-)methyl-O-β-D-glucuronide	**4a**	315.0 > 139.0, 121.2*, 80.0, 113.1	5.64 ± 0.03	9–1250	0.9958	<9 (S/N 405)	9	3.1–19.8	97.4–106.1	3.6–7.5	96.4–109.2
(3,5-dimethylpyrazine-2-yl-)methyl-O-β-D-glucuronide	**4b**	315.0 > 139.0, 121.0*, 80.0	5.92 ± 0.03	9–1250	0.9956	<9 (S/N 92)	9	2.3–11.5	97.1–106.9	4.1–10.4	97.2–107.0
(5,6-dimethylpyrazine-2-yl-)methyl-O-β-D-glucuronide	**4c**	315.0 > 139.0, 121.0*, 80.0, 109.0	6.78 ± 0.03	9–1250	0.9954	<9 (S/N 114)	9	3.4–11.3	97.5–106.9	2.9–5.6	97.6–107.6
(3,6-dimethylpyrazine-2-yl-)methyl-sulfate+	**5a+5b**	216.8 > 80.0, 95.5*	2.62 ± 0.02	9–625	0.9968	<9 (S/N 705)	9	1.9–13.4	96.4–106.7	1.4–3.8	92.3–93.2
(3,5-dimethylpyrazine-2-yl-)methyl-sulfate											
(5,6-dimethylpyrazine-2-yl-)methyl-sulfate	**5c**	216.8 > 80.0, 95.5*	3.57 ± 0.01	9–1250	0.9948	<9 (S/N 565)	9	3.2–7.7	91.6–108.3	2.9–5.2	100.1–100.3
3-d_3_,5-dimethyl-2-pyrazinemethanol	**d_3_-2b**	142.0 > 123.0*, 80.0	4.99 ± 0.04								
3-d_3_,5-dimethylpyrazine-2-carboxylic acid	**d_3_-3b**	156.1 > 110.0, 66.0*	4.90 ± 0.04								
(3-d_3_,5-dimethylpyrazine-2-yl-)methyl-O-β-D-glucuronide	**d_3_-4b**	318.1 > 142.1*, 124.1	5.55 ± 0.03								
(3-d_3_,5-dimethylpyrazine-2-yl-)methyl-sulfate	**d_3_-5b**	218.7 > 79.9, 95.9*	2.59 ± 0.02								

a Quantifier is marked with an asterisk.

b LoD is defined as the lowest standard with SN > 3.

c LloQ is defined as the lowest standard in the calibrated range (*S*/*N* > 10, precision < 15%, accuracy ≥80 to ≤120%).

d Precision and accuracy of back-calculated calibration standards in artificial urine.

e Precision and accuracy of quality controls (supporting Table 1).

#### 2.7.3 Sample Preparation

An aliquot of the standard or authentic sample (urine), respectively (450 μL), was spiked with the mixed IS solution (50 μL) and transferred to an autosampler vial. Aliquots (5 μL) were injected into the UHPLC–MS/MS system. Calibration curves were established by plotting area ratios of the analyte and internal standard versus the respective concentration ratios. Calibration curves were 1/*x* weighed and had *R*^2^ > 0.99.

#### 2.7.4 Data Processing

Processing of raw data and calculation of quantitative data, calibration curves, and QC statistics was done in analyst 1.6.3 (Sciex, Darmstadt, Germany). Quantitative data were analyzed with GraphPad 9.3.0 for Windows (GraphPad Software, San Diego, California USA, www.graphpad.com). Data were tested for outliers and normality/lognormality with the Kolmogorov–Smirnov test. One-way analysis of variance (ANOVA) was done with log-transformed data, Geisser–Greenhouse correction, and Dunnett’s test for multiple comparisons.

## 3 Results and Discussion

Pyrazines are frequently consumed with diet as they are common odorants in thermally processed foods like toasted bread or roasted coffee.^3^ Animal experiments indicate that metabolism primarily involves oxidation and hydroxylation leading to carboxylic acid derivatives as final excretion products. The excretion of phase 2 metabolites has been hypothesized by Adams et al. and Müller and Rappert.^2,8^ In contrast to dimethylpyrazines, little is known about the key flavor odorant TMP.

Aiming at the clarification of excreted metabolites formed from TMP, we synthesized phase 1 and phase 2 metabolites based on literature suggestions^2,4,8^ and one additional deuterium-substituted derivative for each class of analyte to serve as the internal standard. We developed stable isotope dilution assays (SIDAs) to analyze spot urine samples, collected from a human coffee intervention study, by means of UHPLC–MS/MS. The compounds were individually introduced into the mass spectrometer via a syringe for software-assisted tuning of ion sources and path parameters, as well as optimization of energies for collision-induced dissociation (CID) to enable detection in the multiple reaction monitoring (MRM) mode. Glucuronides, pyrazinemethanols, and pyrazine-2-carboxylic acids were easily tuned in positive electrospray (ESI^+^). The pyrazinemethanols **2a–d** produced intense [M + H]^+^ pseudomolecular ions and generated mainly unspecific fragments with the loss of water being the dominant one. The carboxylates (**3a–c**) ionized well in positive electrospray despite the acidic function. Main fragments were [M – H_2_O + H]^+^ and the cleavage of the carboxylic acid group as a loss of formic acid [M – HCOOH + H]^+^. The glucuronide conjugates (**4a–c**) gave abundant [M + H]^+^ pseudomolecular ions in ESI^+^ and delivered daughter ions, indicating the loss of hexuronic acid and a further loss of water. With exception of the glucuronides, we noticed that the MRM traces were relatively noisy despite the two MS-filtering steps in Q1 and Q3. The sulfates **5a–c** were tuned in negative electrospray. The two obtained fragments (*m/z* 80 and 96) indicated cleavage of the sulfate group.

Chromatographic separation was achieved on a 150 mm biphenyl column with acetonitrile and water, each containing 0.1% formic acid and 1 mM ammonium acetate as modifiers. Calibration standards, quality controls, and urine samples were analyzed with a method in positive electrospray for the analytes **2**–**4** and a second method in negative electrospray for the sulfated analytes. Because it was impossible to obtain analyte-free human urine due to the broad occurrence of the odorant 2,3,5-trimethylpyrazine in foods, we used AU prepared from inorganic and organic salts, creatinine, citric acid, and urea to prepare calibration standards and QC.^16^ Area ratios of analyte versus internal standard were plotted against the concentration ratios. The calibrated range was between 9 and 1250 nM, with precision of back-calculated standards <15% and accuracy between 91.7 and 112.6%. QCs showed a precision <13.2% and accuracy between 94.5 and 109.2% (Table 1 and supporting Table 1).

To investigate the contribution of roast coffee consumption on the excretion of metabolites formed from dietary TMP under typical house-hold/real-life conditions, we obtained urine samples from a pilot coffee drinking study recently conducted in our institute. In brief, six healthy participants (3m, 3f) abstained from roast coffee consumption for three consecutive days to reduce internal levels of TMP metabolites. On study day one, the participants brought a morning spot urine sample. Tap water was consumed and a second spot urine sample was collected after 4 h. The following day, the procedure was repeated but instead of tap water, a serving of roast coffee brew (200 mL) was consumed and urine collected. On study day three, the procedure was repeated but instead of one serving of roast coffee brew, two servings were consumed (400 mL) and a final spot urine sample was collected after 2 h. The concentration of TMP in the roast coffee brew determined by SIDA-GC–MS/MS was 4.08 μM (±9.2%, *n* = 3), the ingested amount therefore was 0.82 μmol on day 2 (one coffee serving), and 1.63 μmol on day 3 (two servings). The collected spot urine samples were spiked with the internal standards and analyzed by UHPLC–MS/MS. Concentrations were not adjusted for creatinine as the primary aim of the study was to identify and quantitate key metabolites of TMP rather than to do a quantitative recovery of ingested amounts.

**Figure 3:**
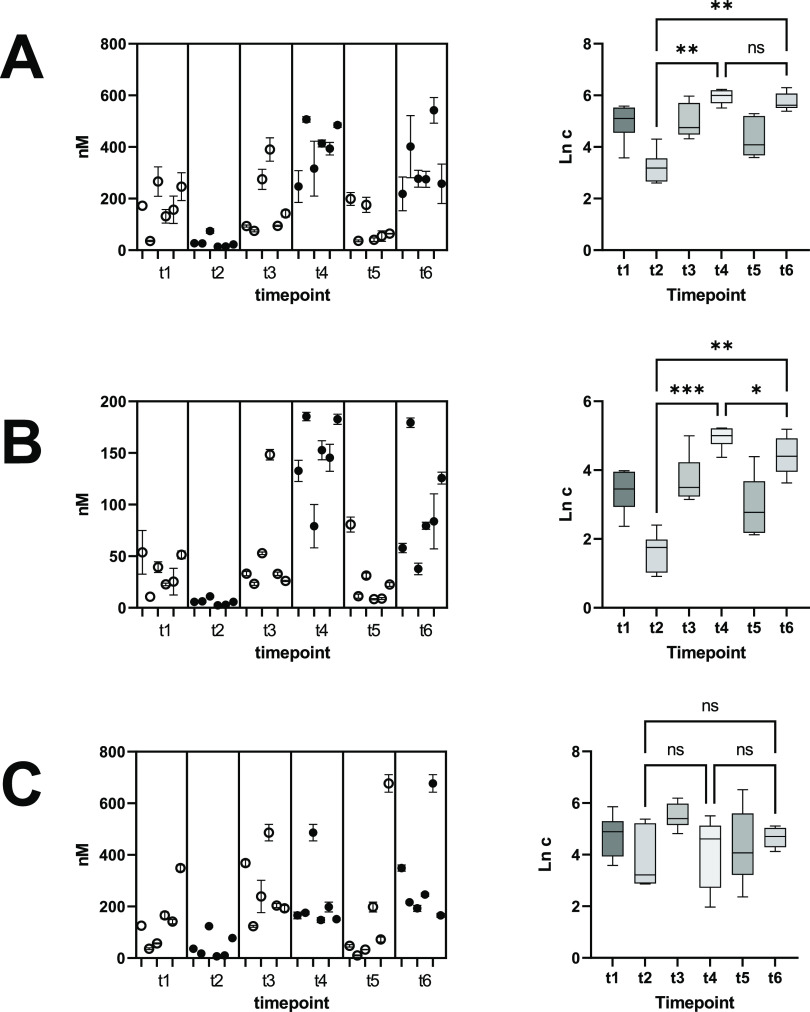
Left part of the figure: Concentration of metabolites in excreted urine on study day 1–3 (*n* = 6 individuals, means ± standard deviation of triplicates). Time points t1, t3, t5 are morning urines (circles), time point t2 is spot urine collected 4 h after tap water, t4 is spot urine collected 4 h after roast coffee brew, and t6 is spot urine collected after 2 h of roast coffee brew (2 servings). Right part of the figure: Data are Ln-transformed prior to ANOVA. One-way ANOVA was done with log-transformed data, Geisser–Greenhouse correction, and Dunnett’s test for multiple comparisons. (A) 3,6-Dimethylpyrazine-2-carboxylic acid (**3a**), (B) 3,5-dimethylpyrazine-2-carboxylic acid (**3b**), (C) 5,6-dimethylpyrazine-2-carboxylic acid (**3c**).

From the selection of metabolite candidates, only the carboxylic acids **3a–c** were in the quantifiable concentration range and showed substantial abundance (Figure 3). In contrast, hydroxylated TMP derivatives **2a–d** were either not detectable (<LoD) or not quantifiable (<LloQ). Phase 2 metabolites **4a–c** were detected in some of the spot urines, but concentrations were below the LloQ. The sulfated analytes **5a** and **b** were detected in all samples, but the concentration was below the LloQ, resulting in negligible concentrations compared to **3a–c**. The derivative **5c** was not detected.

Morning urines (time points t1, t3, t5) contained the metabolites 3,6-dimethylpyrazine-2-carboxylic acid (**3a**), 3,5-dimethylpyrazine-2-carboxylic acid (**3b**), and 5,6-dimethylpyrazine-2-carboxylic acid (**3c**) despite the abstinence from roasted coffee brew. This was most probably due to the omnipresent abundance of TMP (**1**) in heat-processed foods and the dietary restrictions of the study participants being limited to avoidance of roasted coffee. The concentrations of **3a–c** in the morning urines (time points t1, t3, t5, circles in Figure 3) did not vary significantly (*p* > 0.05). Morning urines contained **3a** in the range of 35.7–390.3 nM, **3b** between 8.4 and 148.3 nM and **3c** between 10.7 and 677.0 nM. The concentrations of **3a–c** in the urine collected after water (t2) ingestion were lower ranging between 13.5–74.1, 2.5–11.1, and 79.8–91 nM. After consumption of one serving of coffee (t4), the concentrations rose, ranging between 246.7–507.0 nM (**3a**), 79.1–185.3 nM (**3b**), and 7.2–246 nM (**3c**). Interestingly, two servings of coffee brew (t6) did not result in a significantly elevated concentration of excreted metabolite **3a** (Figure 3A) after 2 h (t6). For **3b**, the concentration was slightly higher after two servings of coffee brew (Figure 3B). The excreted amounts of **3c** apparently originated from other sources than metabolism of TMP because no significant concentration changes were observed (Figure 3).

Pyrazines are odorants in thermally processed foods, like bread crust, roasted meat, and coffee.^3^ Notably, abstinence from roasted coffee alone did not result in the absence of TMP-related metabolites in the morning urines, underlining the broad occurrence in foods. However, after consumption of roast coffee brew, the concentrations in excreted urine significantly rose for 3,6-dimethylpyrazine-2-carboxylic acid (**3a**) and 3,5-dimethylpyrazine-2-carboxylic acid (**3b**). This suggested roasted coffee is a substantial dietary source for TMP, and the two compounds **3a** and **3b** were the major metabolites formed, being excreted in concentrations of >700 nM (Figure 3). Compared to these compounds, the detected traces (<9 nM) of phase 1 metabolites **2a–d** and phase 2 metabolites **4a**, **4b**, and **5a/b** were negligible, being either below the LoD or LloQ.

In conclusion, we report the application of a quantitative UHPLC–MS/MS-based stable isotope dilution assay and results from urine analysis of metabolites of TMP delivered by real-life doses of roasted coffee brew. The study itself, however, has some limitations, as the biosamples were spot urines and therefore did not allow time-resolved analysis and kinetic interpretations, and calculation of excretion rates.^14,23−26^ In contrast to untargeted metabolomics utilizing high-resolution MS-screening aiming at biomarker discovery,^27−29^ the number of participants in the study the biosamples were available from was relatively small with six participants, and substantial interindividual differences in excreted concentrations were detected. Our targeted approach nevertheless succeeded in unambiguous identification and quantification of TMP metabolites as the data show that consumption of roasted coffee brew leads to elevated concentrations of TMP metabolites **3a** and **3b**. However, it is apparent that coffee is not the only contributor to their abundance.

Future pharmacokinetic investigations on the formation of carboxylated TMP metabolites will benefit from the study participants following a strict pyrazine-free diet during the run-in period, taking noncoffee sources for TMP into account, to minimize initial metabolite abundance. Normalizing determined metabolite concentrations to individual creatinine concentrations will further help minimize the spread in the analytical data. Despite these limitations, the data suggest that the formation of phase 1 metabolites **3a–c** was the preferred metabolization route for of TMP, being in line with previous results on other pyrazine derivatives,^14^ and no substantial abundance of phase 2 metabolites was recorded.

## Supporting Information Available

Supporting InformationPrecision and accuracy of the analysis of putative metabolites of 2,3,5-trimethylpyrazine in quality controls (supporting Table 1); concentrations (means ± standard deviation) in human spot urine (supporting Table 2); chromatographic purification of all synthesized compounds (Figures S1–S4); NMR spectra of all synthesized compounds (Figures S5–S41); and MS/MS spectra (product ions) of all synthesized compounds (Figures S42–S58) (PDF)

## Abbreviations

AU: artificial urine
DCM: dichloromethane
ESI: electrospray ionization
FEMA: flavor extract manufacturing association
GRAS: generally recognized as safe
HPLC: high-performance liquid chromatography
IS: internal standard
LC-TOF-MS: liquid chromatography-time-of-flight mass spectrometry
MS: mass spectrometry
MS/MS: tandem mass spectrometry
NMR: nuclear magnetic resonance spectroscopy
QC: quality control
RP18: reversed phase C18
SIDA: stable isotope dilution analysis
TMP: 2,3,5-trimethylpyrazine
UHPLC: ultrahigh-performance liquid chromatography
