# Supplementary material for: Metabolites of Key Flavor Compound 2,3,5-Trimethylpyrazine in Human Urine
Source: J Agric Food Chem. 2022 Nov 18;70(48):15134–42. doi: 10.1021/acs.jafc.2c06418 (PMC9733599; doi:10.1021/acs.jafc.2c06418)
Supplement: Supplementary file 1 — jf2c06418_si_001.pdf [file jf2c06418_si_001.pdf]

Supporting Information

Metabolites of key flavor compound 2,3,5-trimethylpyrazine in human urine

Dong Liang[1,2,#], Sebastian Dirndorfer[1], Veronika Somoza[1], Dietmar Krautwurst[1], Roman Lang[1,*], and Thomas Hofmann[1,2]

[1] Leibniz Institute for Food Systems Biology at the Technical University Munich, Lise-Meitner-Str. 34, 85354 Freising, Germany.

[2] Chair for Food Chemistry and Molecular Sensory Science, Technical University Munich, Lise-Meitner-Str. 34, 85354 Freising, Germany.

[#] Present Address of Dong Liang: School of Agriculture, Sun Yat-sen University, Shenzhen, 518107, China

[*] corresponding author: Roman Lang, r.lang.leibniz-lsb@tum.de, orcid.org/0000-0003-0610-7186, Lise-Meitner-Str. 34, 85354 Freising, Germany, tel. +49 8161 2978.

Supporting table 1. Precision and accuracy of the analysis of putative metabolites of 2,3,5-trimethylpyrazine in quality controls.

Analyte		Found (nM)	RSD (%)	Accuracy (%)	Found (nM)	RSD (%)	Accuracy (%)	Found (nM)	RSD (%)	Accuracy (%)
3,6-dimethyl-2-pyrazinemethanol	**2a**	307.9±11.0	3.6	98.4	295.8±23.6	7.9	94.5	316.2±14.9	4.7	101.0
3,5-dimethyl-2-pyrazinemethanol	**2b**	307.2±18.9	6.2	98.2	322.9±16.4	5.1	103.2	335.1±17.1	5.1	107.1
5,6-dimethyl-2-pyrazinemethanol	**2c**	316.4±11.1	3.5	101.0	313.4±10.8	3.4	100.1	331.2±7.4	2.2	105.8
3,5,6-trimethylpyrazine-2-ol	**2d**	324.1±35.3	10.9	103.5	307.4±27.1	8.8	98.2	335.5±42.4	12.6	107.2
3,6-dimethylpyrazine-2-carboxylic acid	**3a**	321.9±24.1	7.5	102.8	311.3±19.2	6.1	99.5	326.0±27.3	8.4	104.2
3,5-dimethylpyrazine-2-carboxylic acid	**3b**	314.4±41.4	13.2	96.9	320.1±38.0	11.9	98.7	328.4±22.9	6.9	101.3
5,6-dimethylpyrazine-2-carboxylic acid	**3c**	315.9±21.3	6.7	100.9	320.5±22.5	7.0	102.4	325.3±34.8	10.7	103.9
(3,6-dimethylpyrazine-2-yl-)methyl-O-β-D-glucuronide	**4a**	310.5±±23.3	7.5	99.2	301.6±10.7	3.6	96.4	341.9±26.7	7.8	109.2
(3,5-dimethylpyrazine-2-yl-)methyl-O-β-D-glucuronide	**4b**	319.7±23.8	7.5	102.2	304.3±12.6	4.1	97.2	334.9±34.9	10.4	107.0
(5,6-dimethylpyrazine-2-yl-)methyl-O-β-D-glucuronide	**4c**	310.7±10.8	3.5	99.3	305.5±17.1	5.6	97.6	336.7±10.0	2.9	107.6
(3,6-dimethylpyrazine-2-yl-)methyl-sulfate+ (3,5-dimethylpyrazine-2-yl-)methyl-sulfate	**5a+b**	290.6±5.7	1.9	92.8	288.9±11.0	3.8	92.3	291.9±4.2	1.4	93.2
(5,6-dimethylpyrazine-2-yl-)methyl-sulfate	**5c**	313.2±12.1	3.9	100.1	313.8±16.3	5.2	100.3	313.7±9.1	2.9	100.2

Quality controls were prepared in triplicates. Data are means±standard deviation of n=6 injections.

Supporting table 2. Concentrations (means±standard deviation) in human spot urine.

	t1	t2	t3	t4	t5	t6
**2a**	0.9 – 7.4 (4.1±4.6), n=2 [a]	0.8 – 3.6 (2.1±1.1), n=4 [a]	0.1 – 2.4 (1.3±0.8), n=5 [a]	0.5 – 2.4 (1.3±0.8), n=4 [a]	0.5 – 9.7 (3.5±4.2), n=4 [a]	0.7 – 2.7 (1.9±0.8), [illegible]
**2b**						
**2c**						
**2d**	1.8 – 5.9 (3.6±1.7), n=4 [a]		0.4 – 6.1 (1.8±2.4), n=5 [a]	1.1 – 5.9 (3.4±1.7), n=6 [a]	0.4 – 7.1 (3.5±3.0), n=4 [a]	0.7 – 11.6 (4.5±4.0) [illegible]
**3a**	35.7 – 266.0 (168.1±83.3), n=6	13.5 – 74.1 (29.7±22.5) , n=6	74.8 – 390.3 (178.4±126.8) , n=6	246.7 – 507.0 (393.6±99.2) , n=6	36.3 – 198.7 (95.0±72.4) , n=6	218.3 – 542.0 (328.[illegible] n=6
**3b**	10.7 – 53.7 (33.9±17.1), n=6	2.5 – 11.1 (5.7±3.1), n=6	23.2 – 148.3 (52.8±47.9), n=6	79.1 – 185.3 (146.3±38.9), n=6	8.4 – 80.7 (27.2±27.7), n=6	37.7 – 179.3 (93.9±[illegible]
**3c**	36.1 – 349.0 (145.7±111.4), n=6	17.6 – 216.3 (79.8±91.0), n=6	123.3 – 486 (268.9±133.7), n=6	7.2 – 246.0 (103.9±88.5), n=6	10.7 – 677.0 (173.1±255.5), n=6	61.8 – 165.7 (113.6[illegible]
**4a**			4.2 – 4.9 (4.5±0.3), n=3 [a]	0.0 – 4.9 (1.8±2.2), n=4 [a]	0.3 – 6.9 (2.3±2.7), n=5 [a]	0.2 – 5.3 (1.7±2.5), [illegible]
**4b**	9.9, n=1		4.5 – 11.2 (8.6±3.6), n=3	2.1 – 39.9 (15.2±15.7), n=4	9.2 n=1	3.2 – 22.3 (11.0±7.6[illegible]
**4c**						
**5a+5b**	[b]	[b]	[b]	[b]	[b]	[b]
**5c**						

[a] below LloQ; [b] qualifier and quantifier detected but calculated concentration too small; empty cells = no peak, < LoD

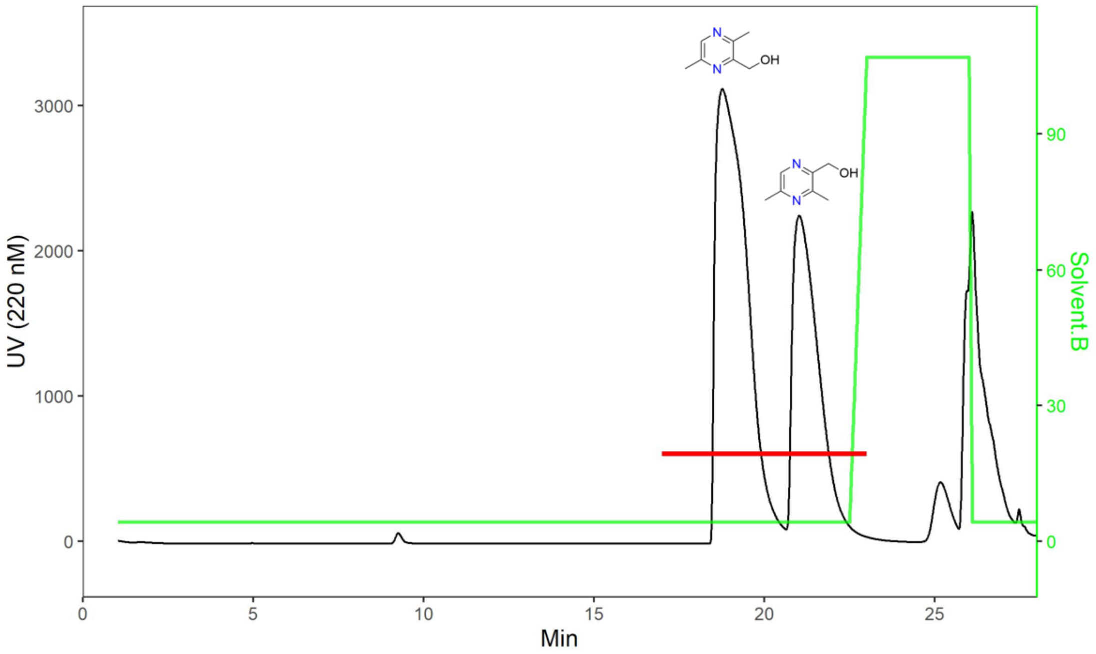

Figure S1. Preparative HPLC separation of trimethylpyrazine hydroxide isomers (**2a** and **2b**).

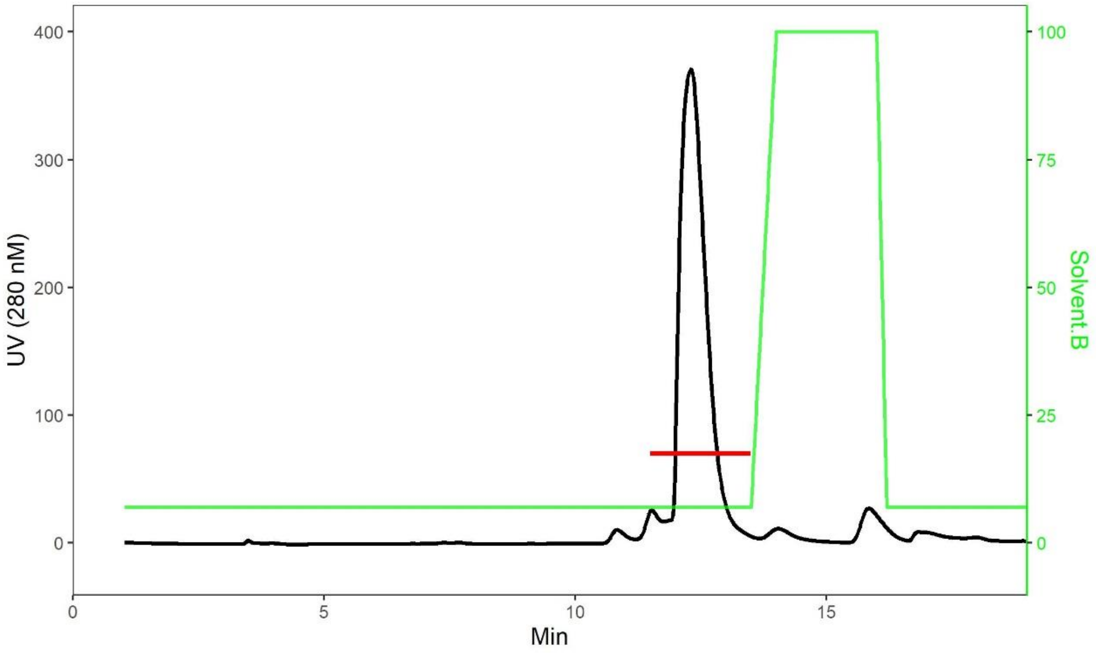

Figure S2. Preparative HPLC separation of trimethylpyrazine glucuronide.

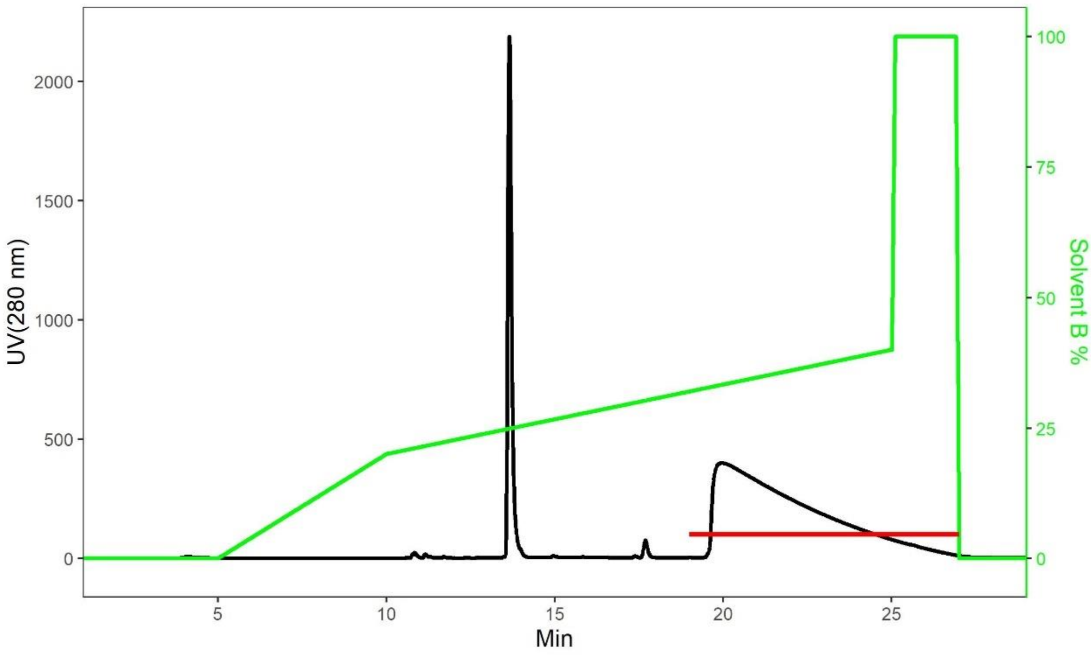

Figure S3. Preparative HPLC separation of trimethylpyrazine sulfate.

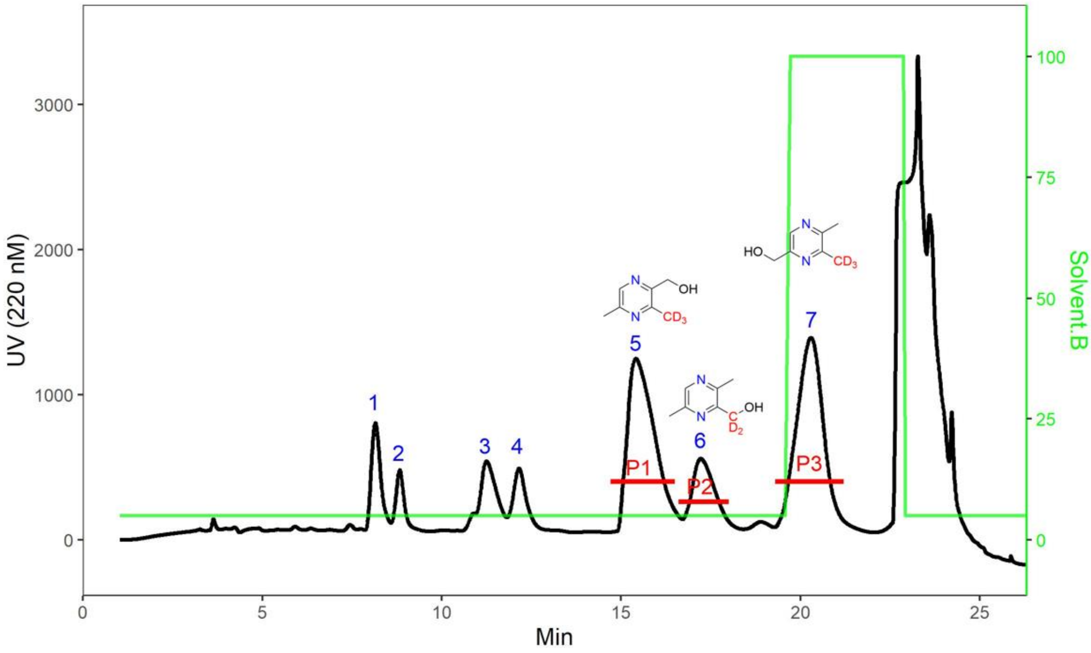

Figure S4. Preparative HPLC separation of trimethylpyrazine-d_3 hydroxide.

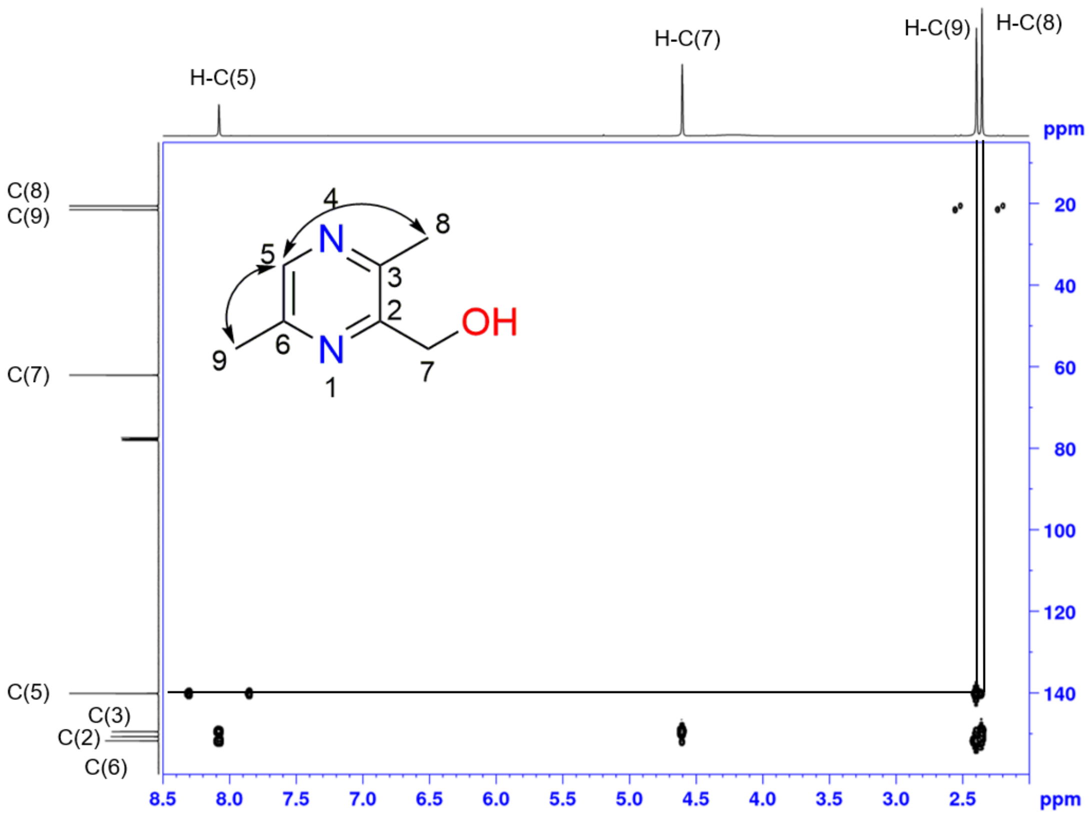

Figure S5. HMBC (400 MHz, 100 MHz, $CDCl_3$) of 3,6-dimethyl-2-pyrazinemethanol (**2a**).

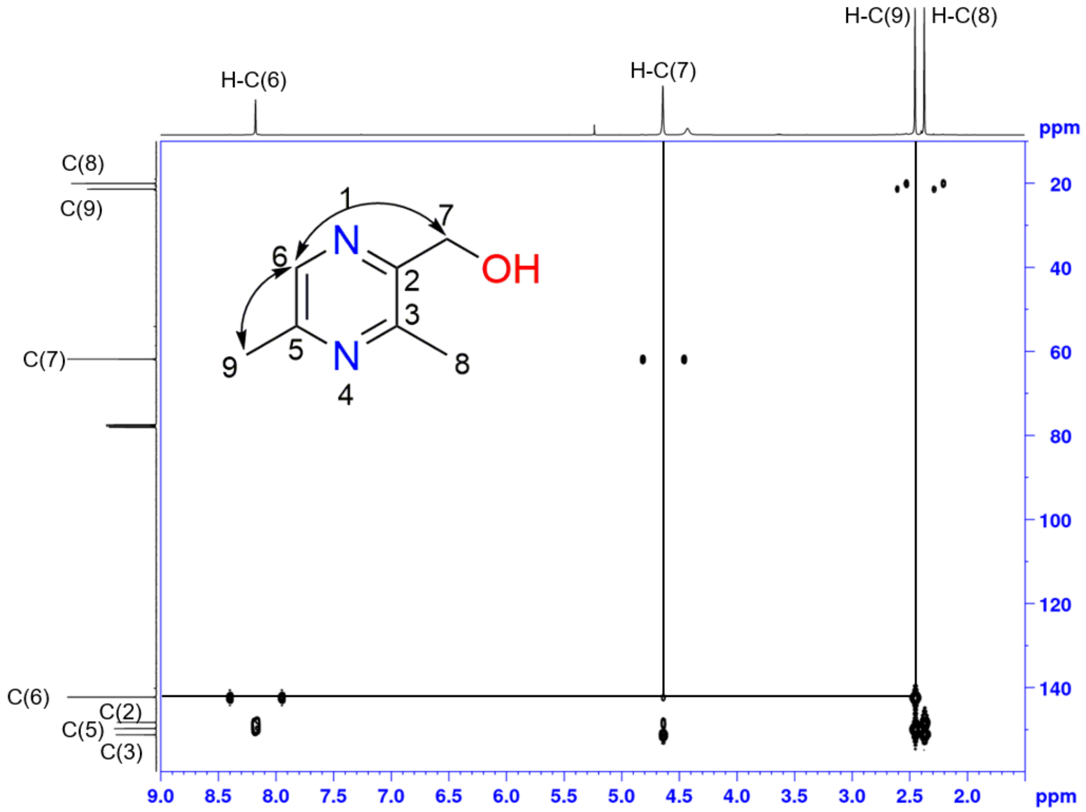

Figure S6. HMBC (400 MHz, 100 MHz, $CDCl_3$) of 3,5-dimethyl-2-pyrazinemethanol (**2b**).

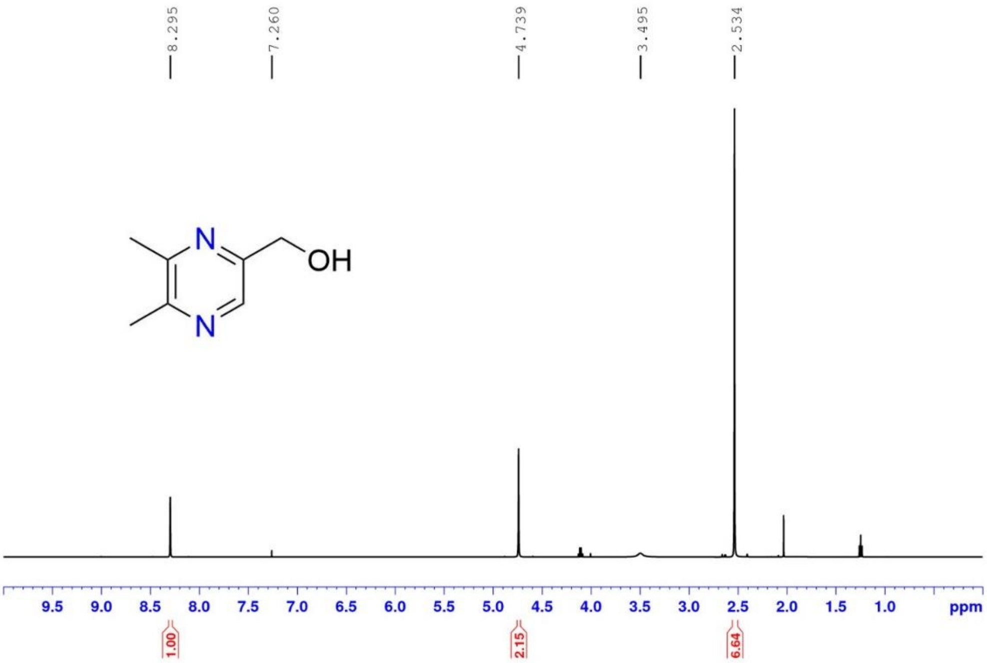

Figure S7. ^{1}H-NMR (500 MHz, $CDCl_3$) of *5,6-dimethyl 2-pyrazinemethanol (**2c**)*

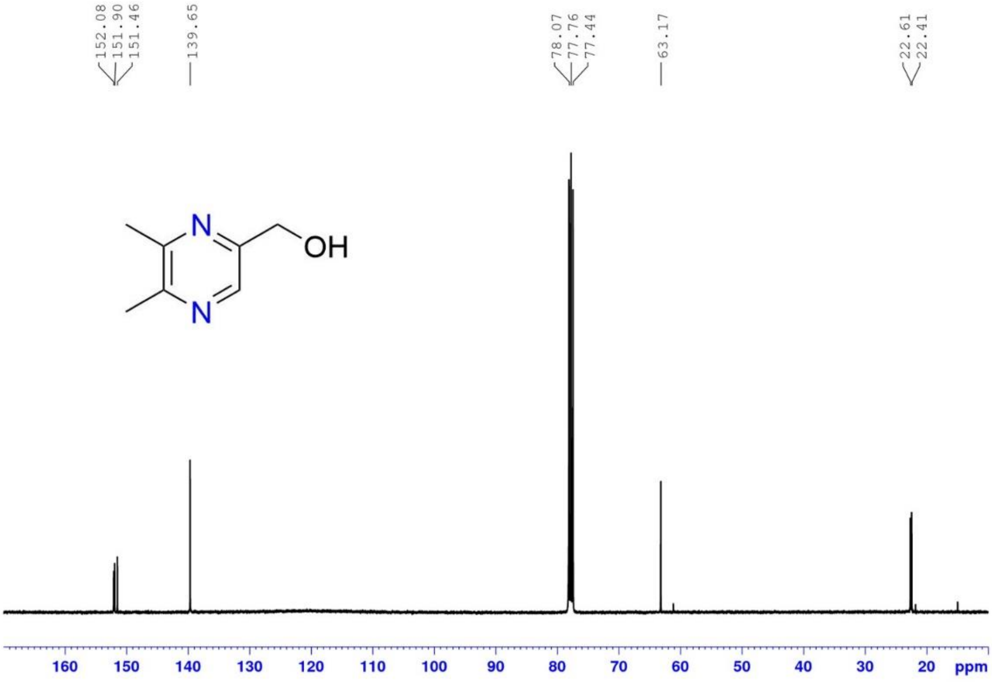

Figure S8. ^{13}C-NMR (125 MHz) of *5,6-dimethyl 2-pyrazinemethanol (**2c**)*

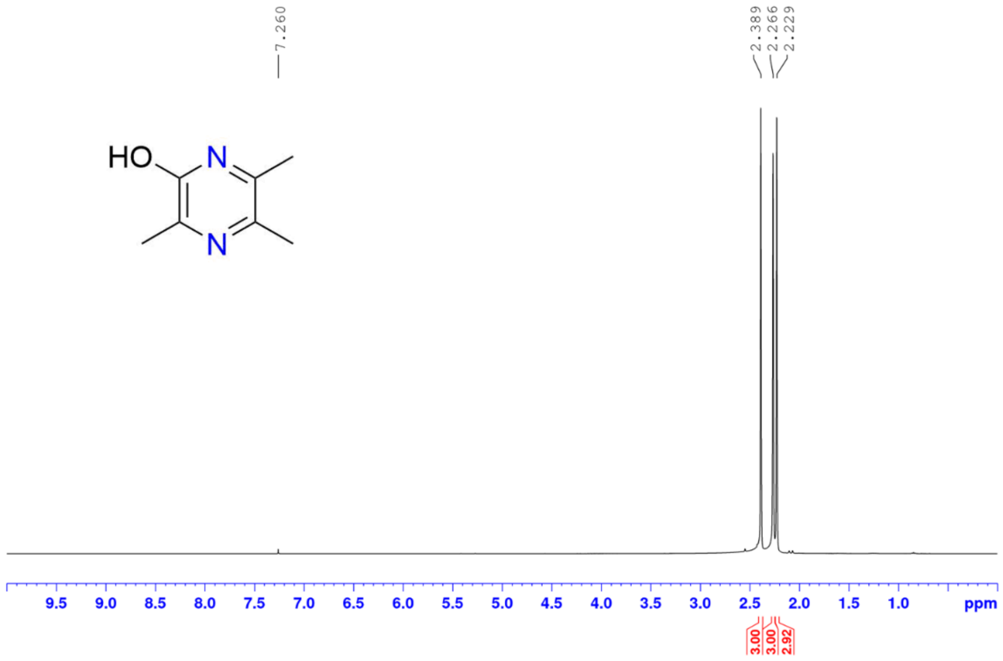

Figure S9. ^{1}H-NMR (400 MHz, $CDCl_3$) of *3,5,6-trimethylpyrazin-2-ol (**2d**)*

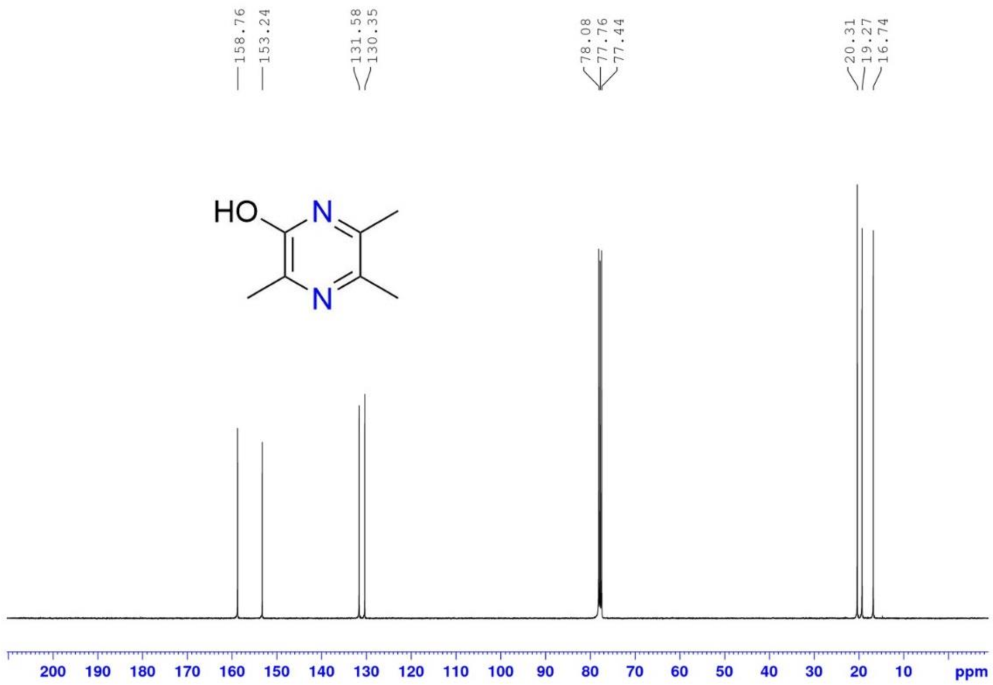

Figure S10. ^{13}C-NMR (400 MHz, $CDCl_3$) of *3,5,6-trimethylpyrazin-2-ol (**2d**)*

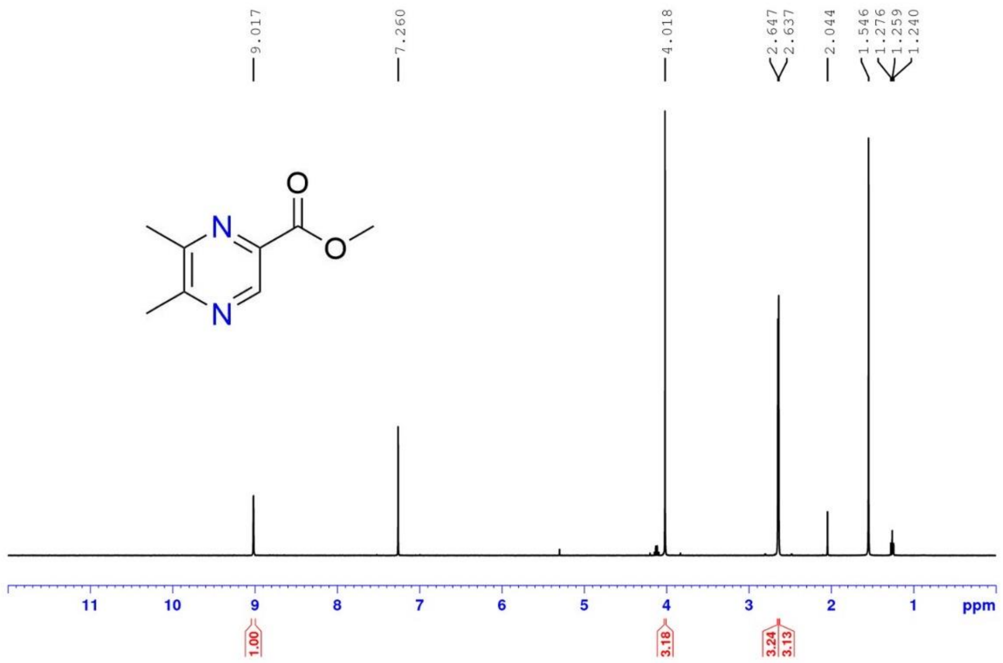

Figure S11. ^{1}H-NMR of Methyl 5,6-dimethylpyrazine-2-carboxylate

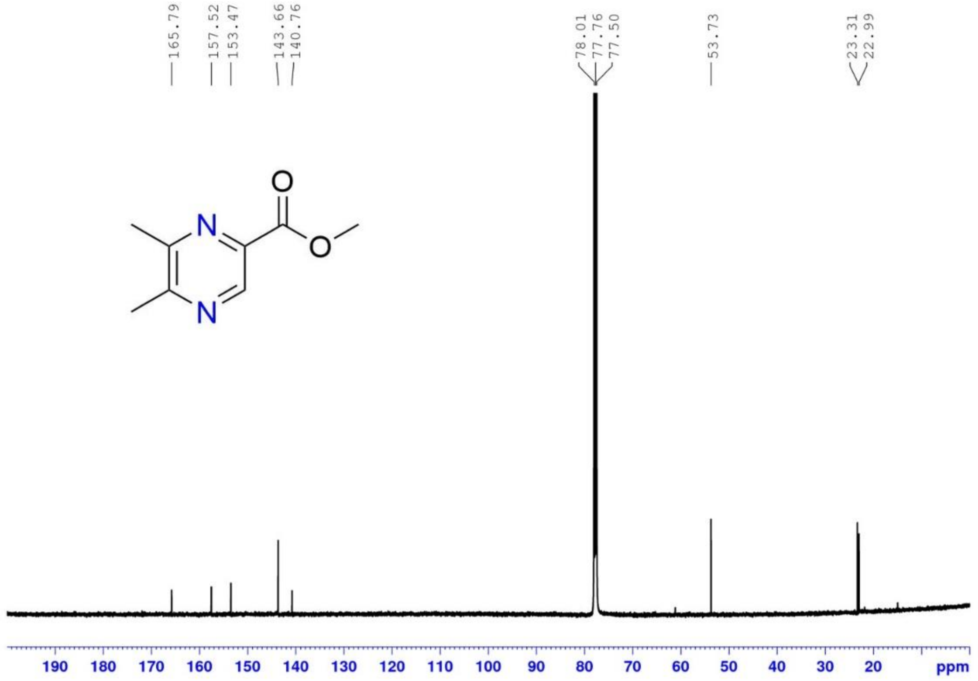

Figure S12. ^{13}C NMR of Methyl 5,6-dimethylpyrazine-2-carboxylate

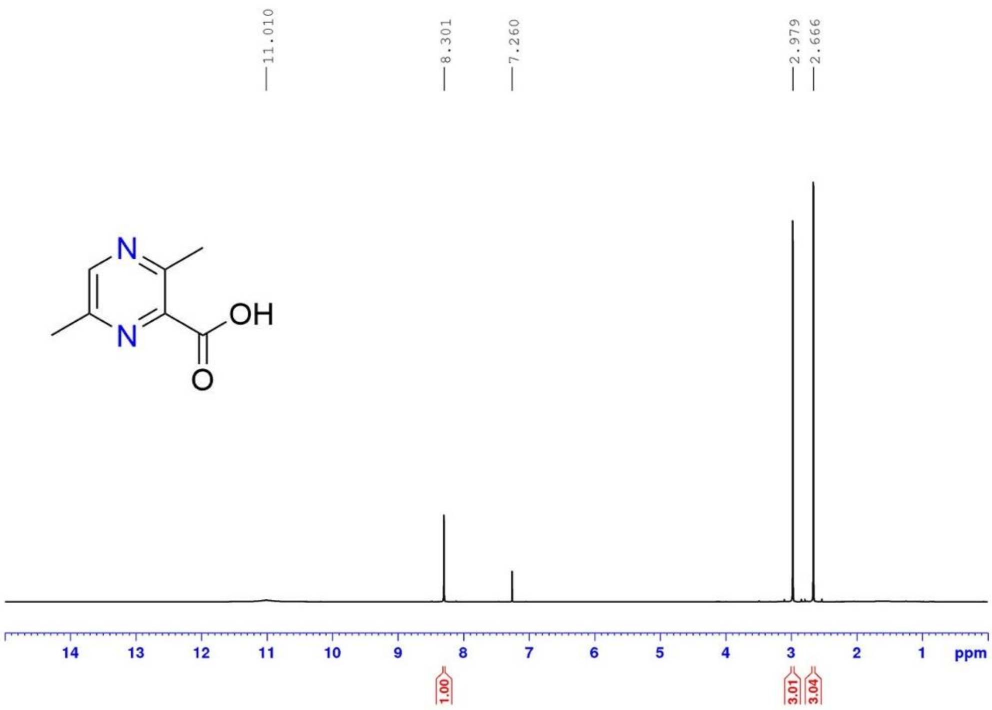

Figure S13. ^{1}H-NMR (500 MHz, $CDCl_3$) of *3,6-dimethylpyrazine-2-carboxylic acid (**3a**)*

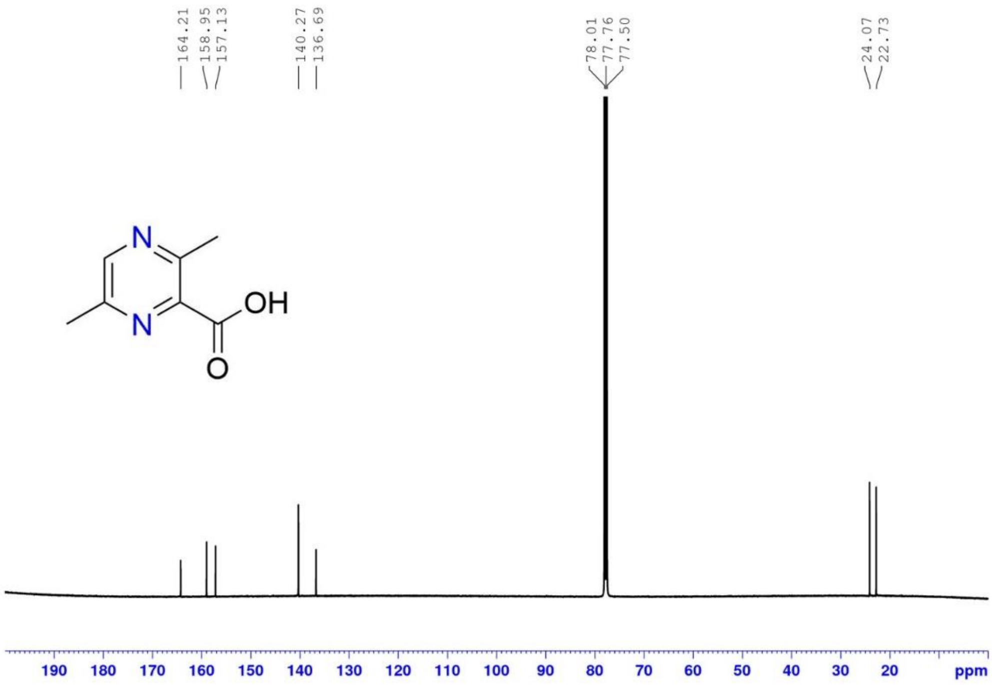

Figure S14. ^{13}C-NMR (125 MHz, $CDCl_3$) of *3,6-dimethylpyrazine-2-carboxylic acid (**3a**)*

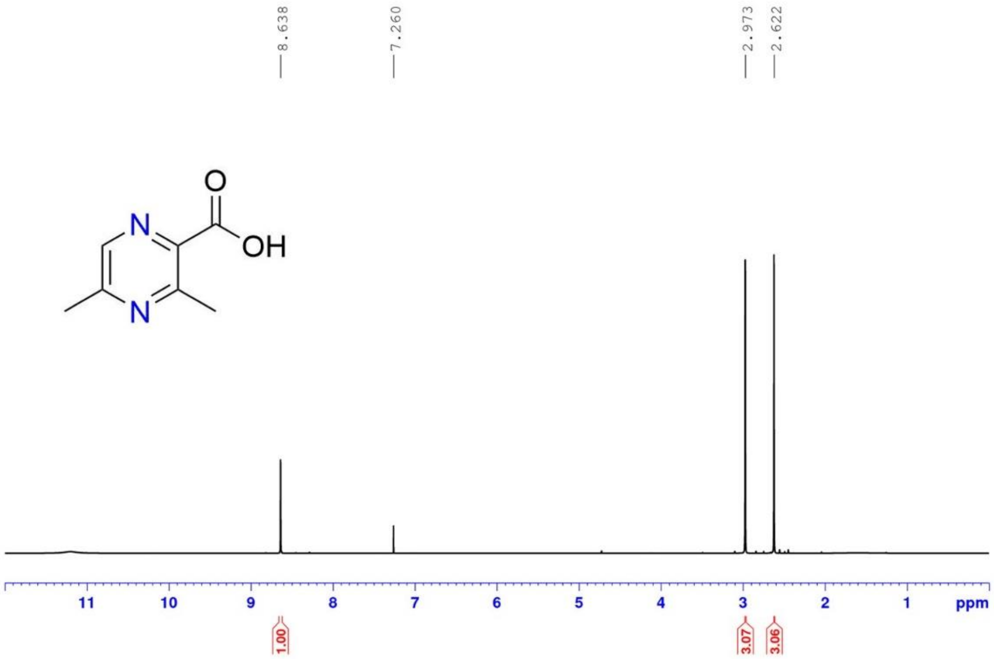

Figure S15. ^{1}H NMR (500 MHz, $CDCl_3$) of 3,5-dimethylpyrazine-2-carboxylic acid (**3b**)

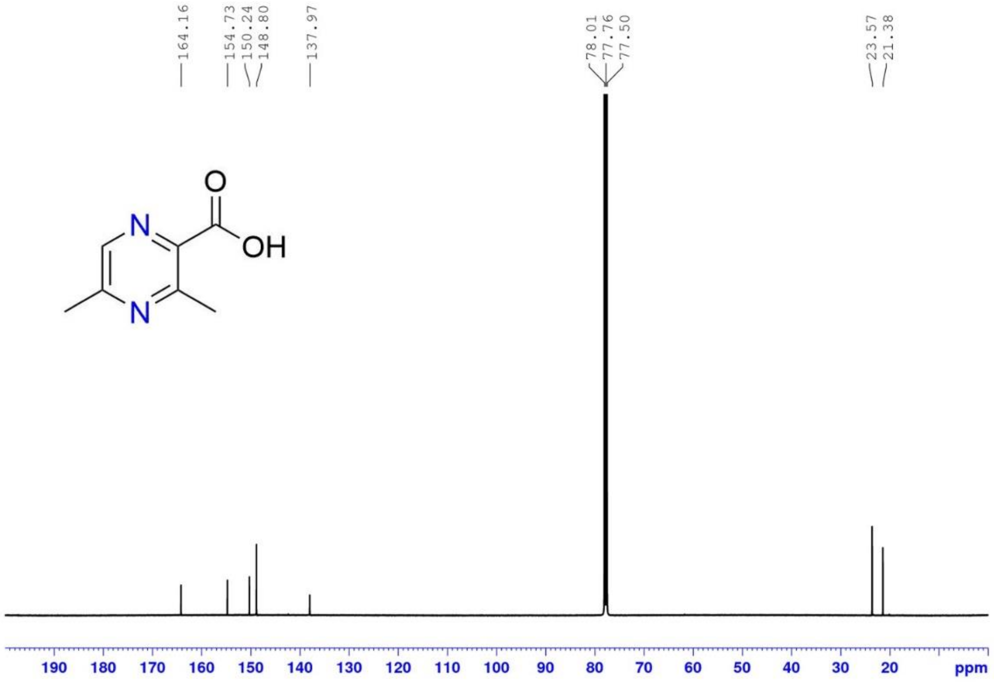

Figure S16. ^{13}C NMR (125 MHz, $CDCl_3$) of 3,5-dimethylpyrazine-2-carboxylic acid (**3b**)

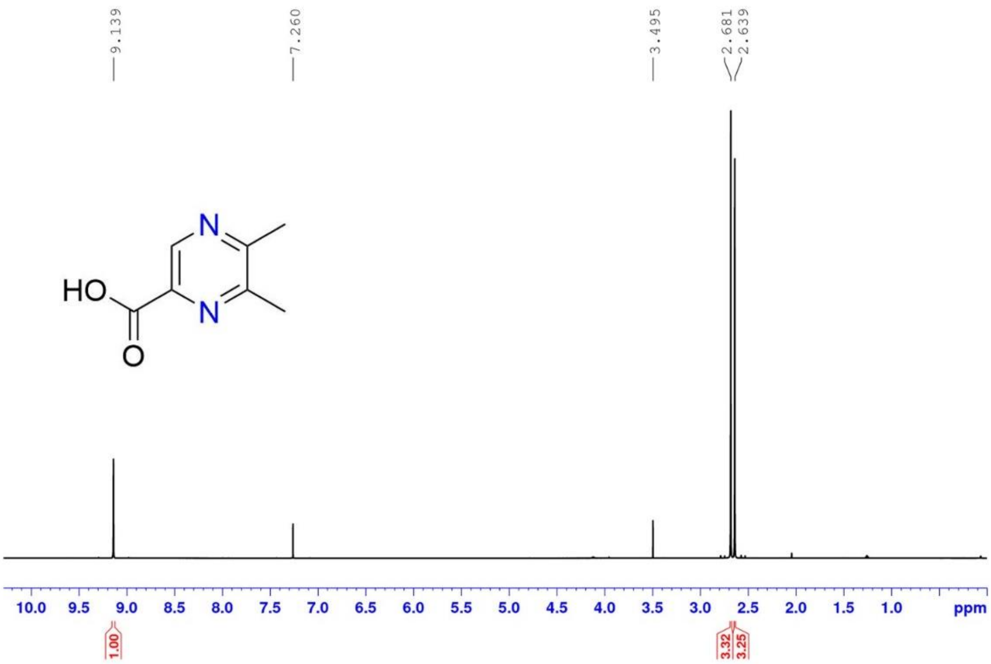

Figure S17. ^{1}H NMR (600 MHz, $CDCl_3$) of 5,6-dimethylpyrazine-2-carboxylic acid (**3c**)

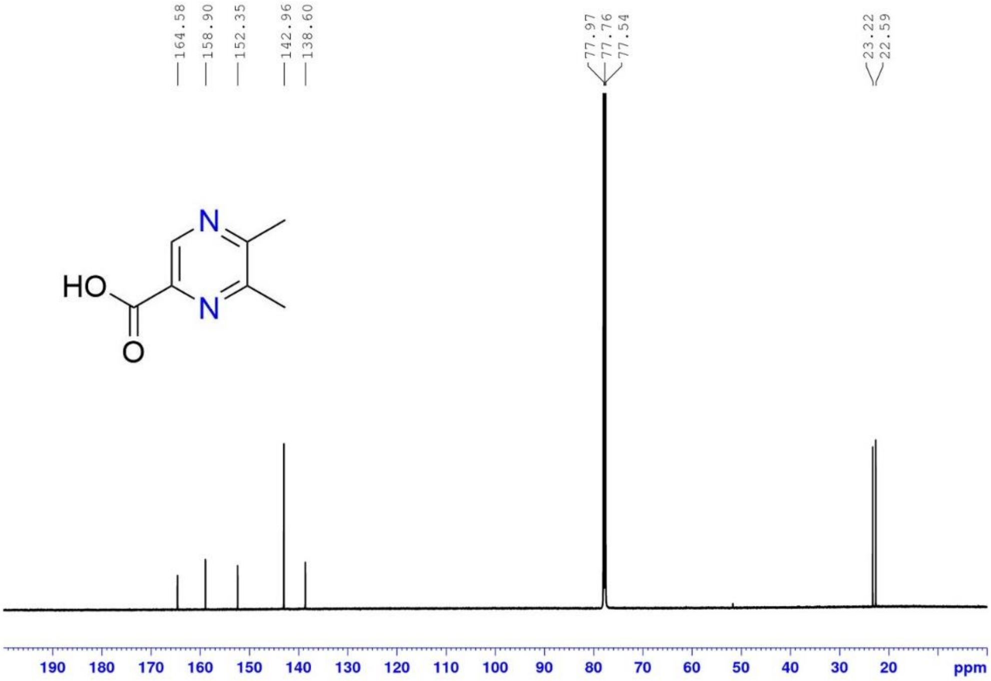

Figure S18. ^{13}C NMR (150 MHz, $CDCl_3$) of 5,6-dimethylpyrazine-2-carboxylic acid (**3c**)

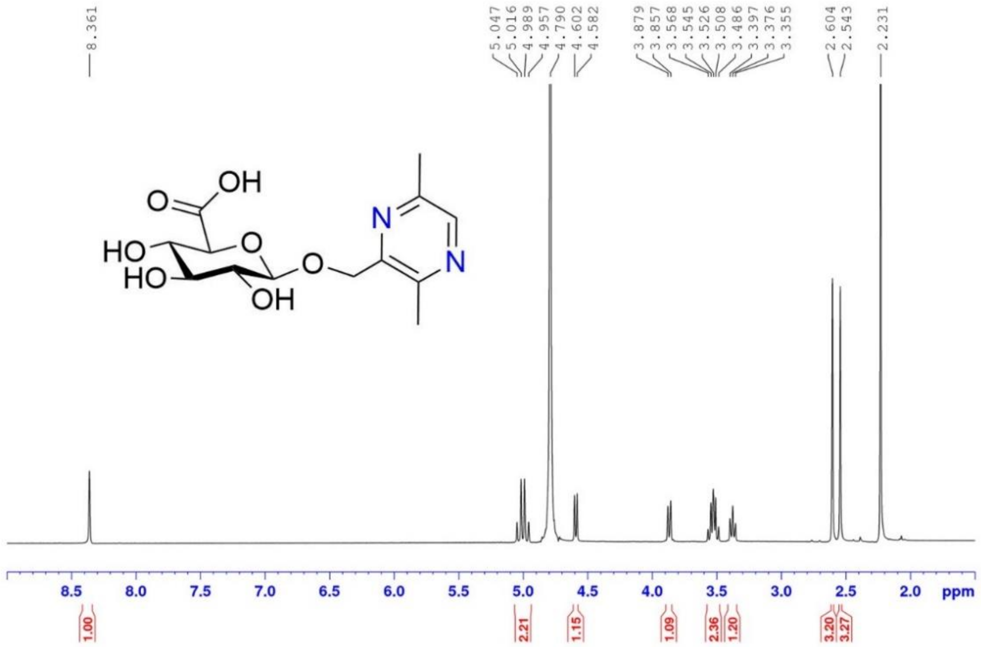

Figure S19. ^{1}H NMR (400 MHz, D_2O) of *(3,6-dimethylpyrazine-2-yl-)methyl-O-β-D-glucuronide (**4a**)*

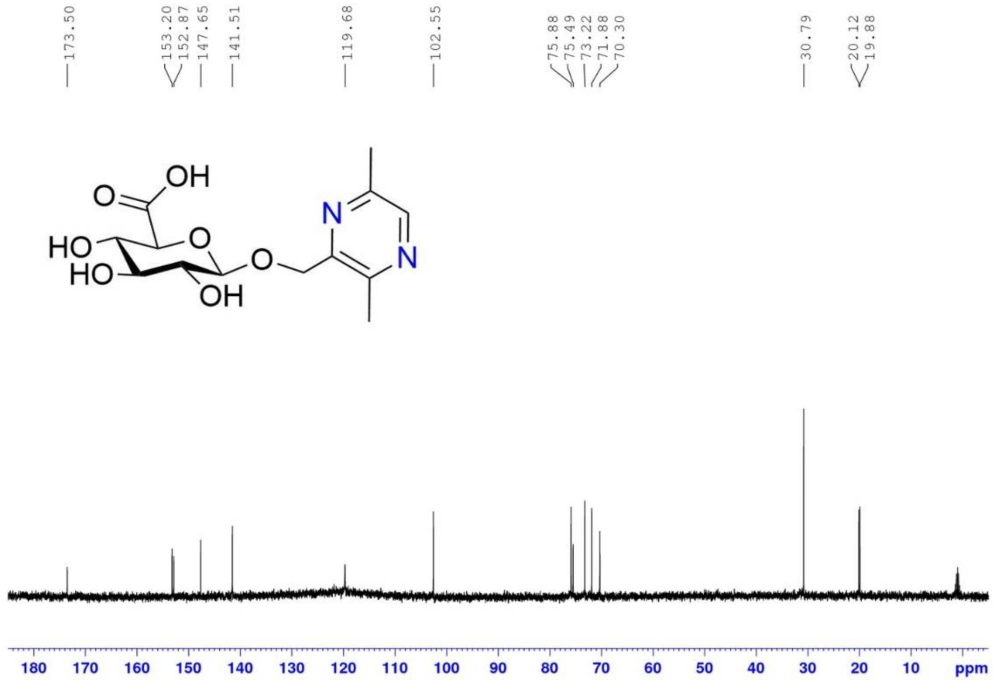

Figure S20. ^{13}C NMR (100 MHz, D_2O) of *(3,6-dimethylpyrazine-2-yl-)methyl-O-β-D-glucuronide (**4a**)*

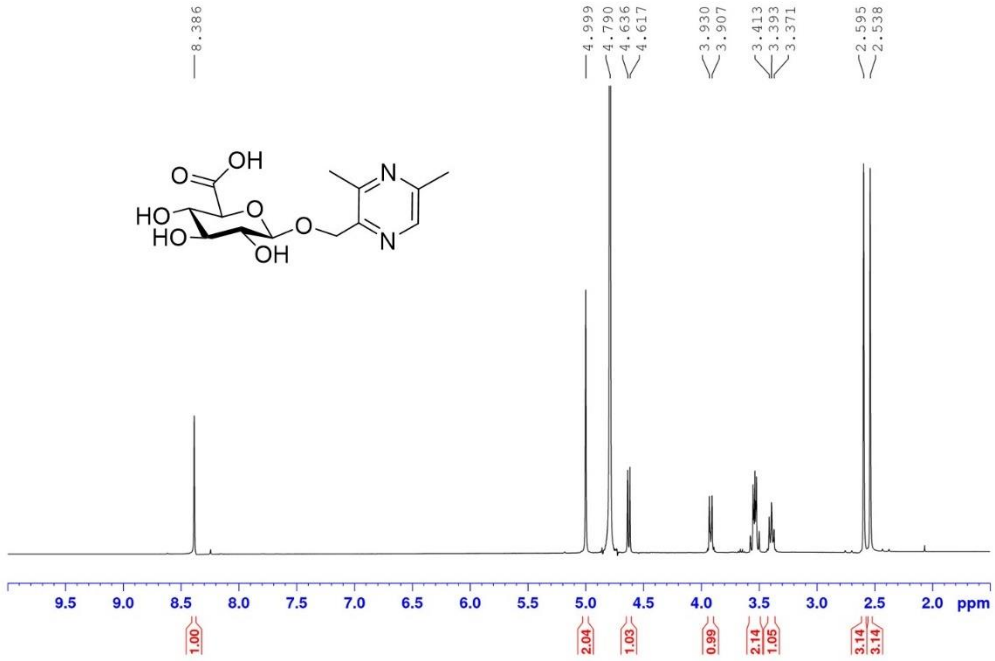

Figure S21. ^{1}H NMR (400 MHz, D_2O) of *(3,5-dimethylpyrazine-2-yl-)methyl-O-β-D-glucuronide (**4b**)*

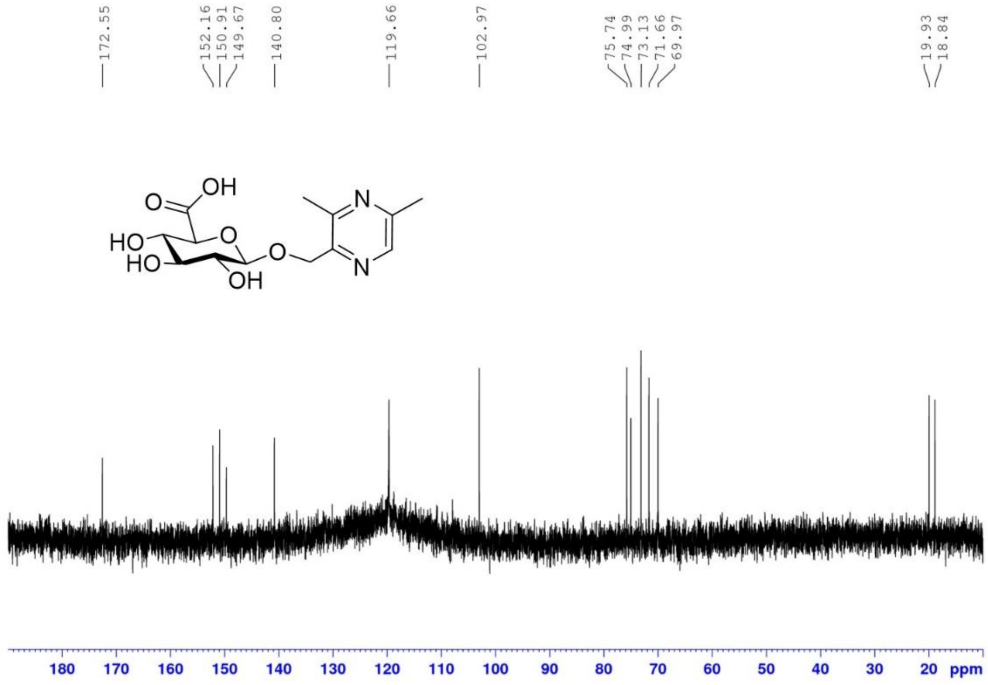

Figure S22. ^{13}C NMR (100 MHz, D_2O) of *(3,5-dimethylpyrazine-2-yl-)methyl-O-β-D-glucuronide (**4b**)*

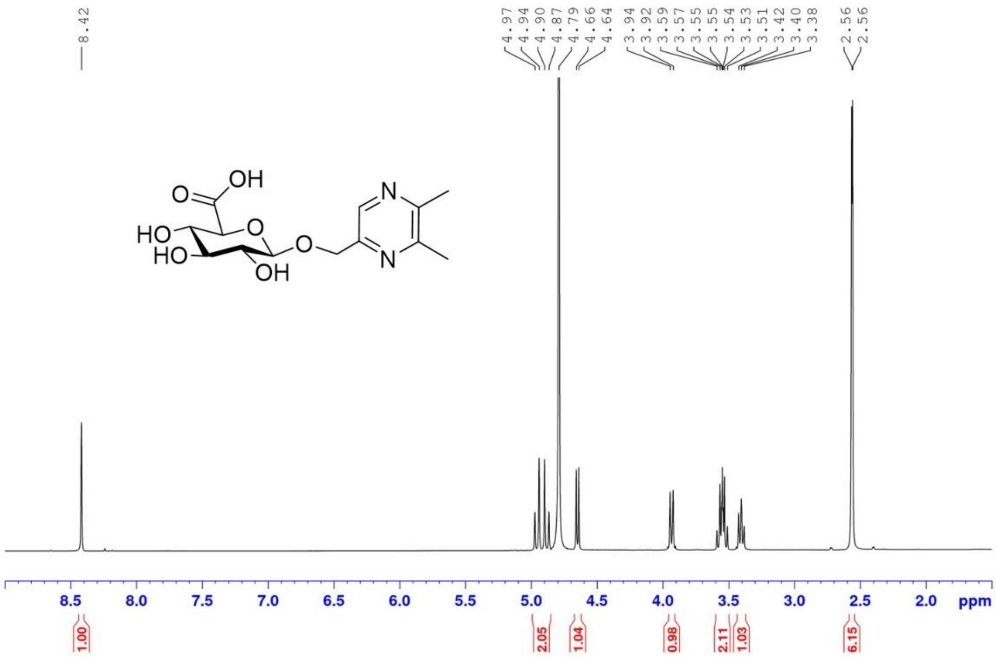

Figure S23. ^{1}H NMR (400 MHz, D_2O) of *(5,6-dimethylpyrazine-2-yl-)methyl-O-β-D-glucuronide (**4c**)*

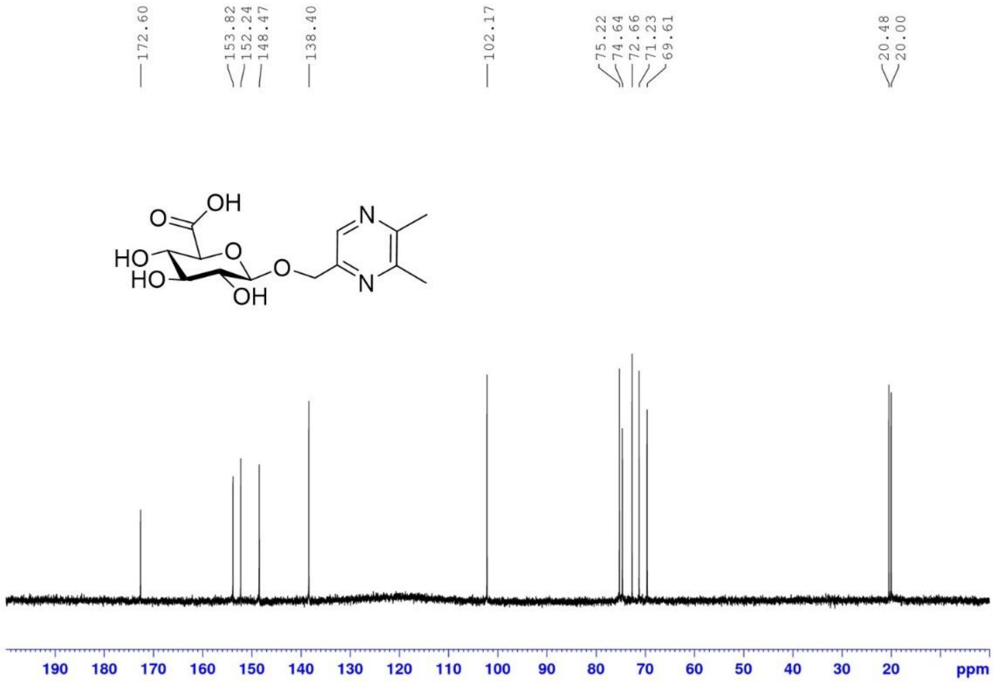

Figure S24. ^{13}C NMR (100 MHz, D_2O) of *(5,6-dimethylpyrazine-2-yl-)methyl-O-β-D-glucuronide (**4c**)*

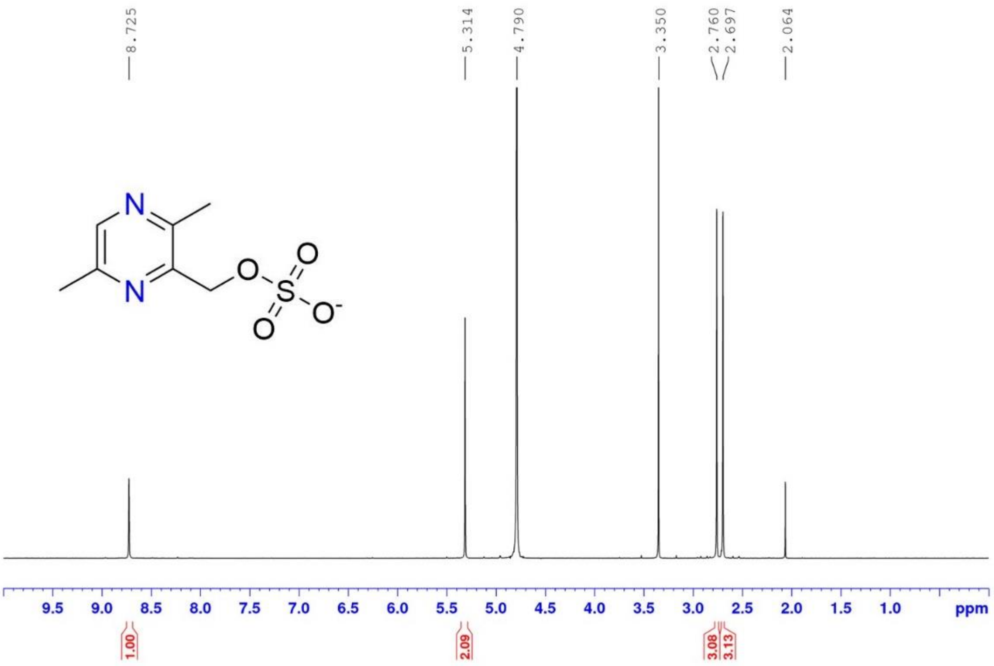

Figure S25. ^{1}H NMR (400 MHz, D_2O) of *(3,6-dimethylpyrazine-2-yl-)methyl-sulfate (**5a**)*

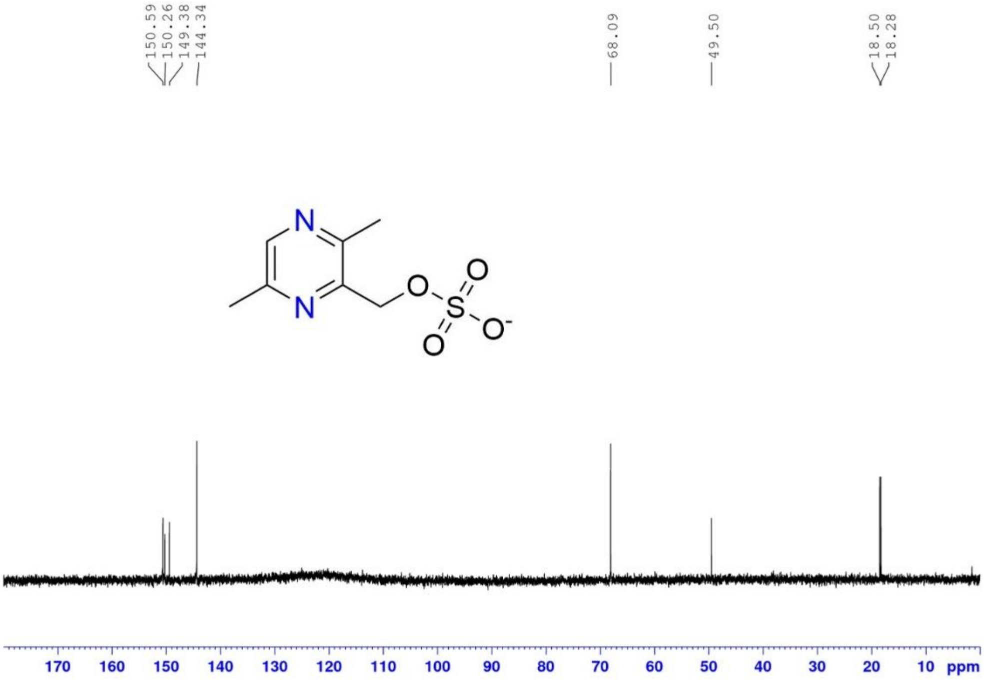

Figure S26. ^{13}C NMR (100 MHz, D_2O) of *(3,6-dimethylpyrazine-2-yl-)methyl-sulfate (**5a**)*

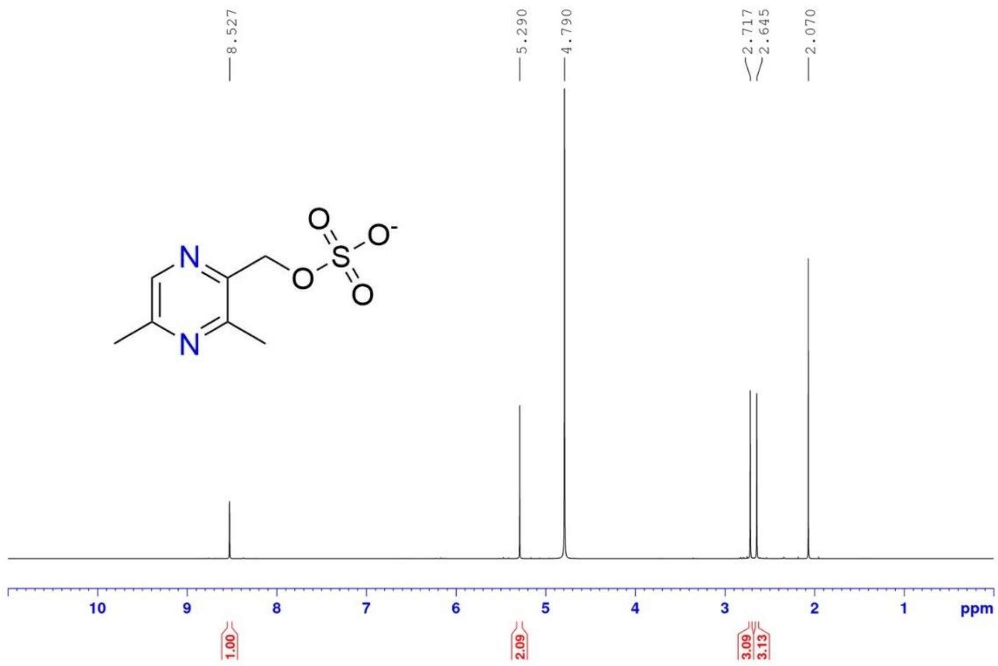

Figure S27. ^{1}H NMR (600 MHz, D_2O) of *(3,5-dimethylpyrazine-2-yl-)methyl-sulfate (**5b**)*

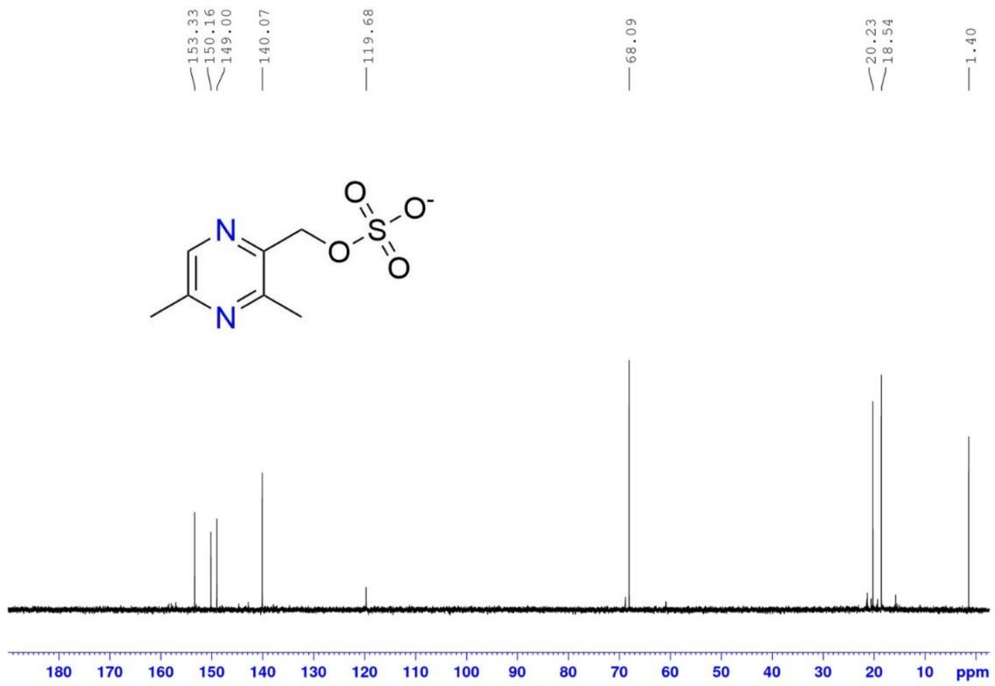

Figure S28. ^{13}C NMR (150 MHz, D_2O) of *(3,5-dimethylpyrazine-2-yl-)methyl-sulfate (**5b**)*

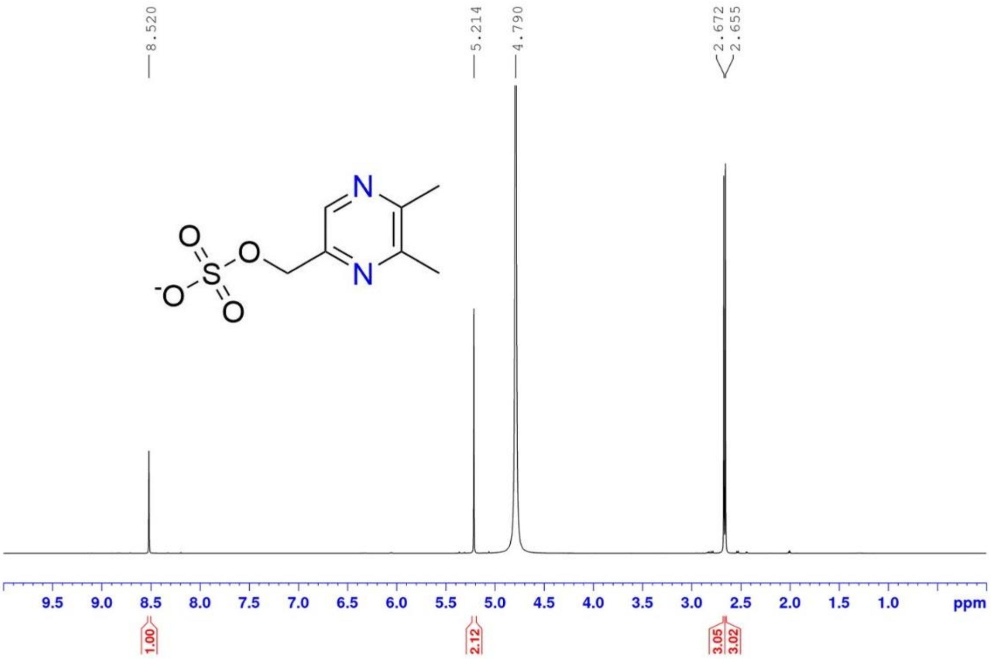

Figure S29. ^{1}H NMR (500 MHz, D_2O) of *(5,6-dimethylpyrazine-2-yl-)methyl-sulfate (**5c**)*

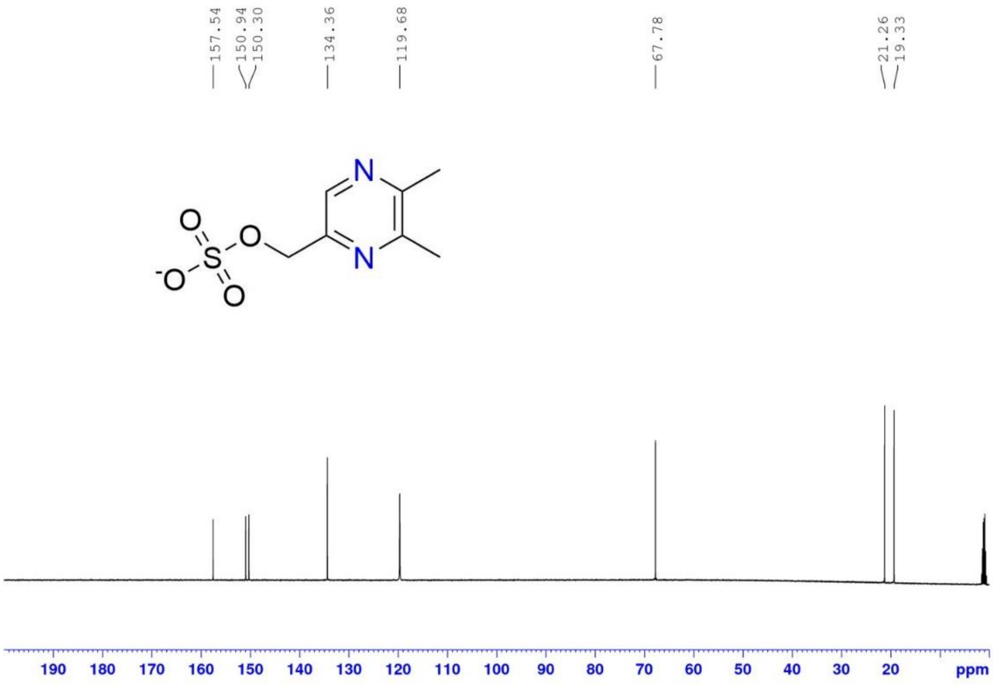

Figure S30. ^{13}C NMR (125 MHz, D_2O) of *(5,6-dimethylpyrazine-2-yl-)methyl-sulfate (**5c**).*

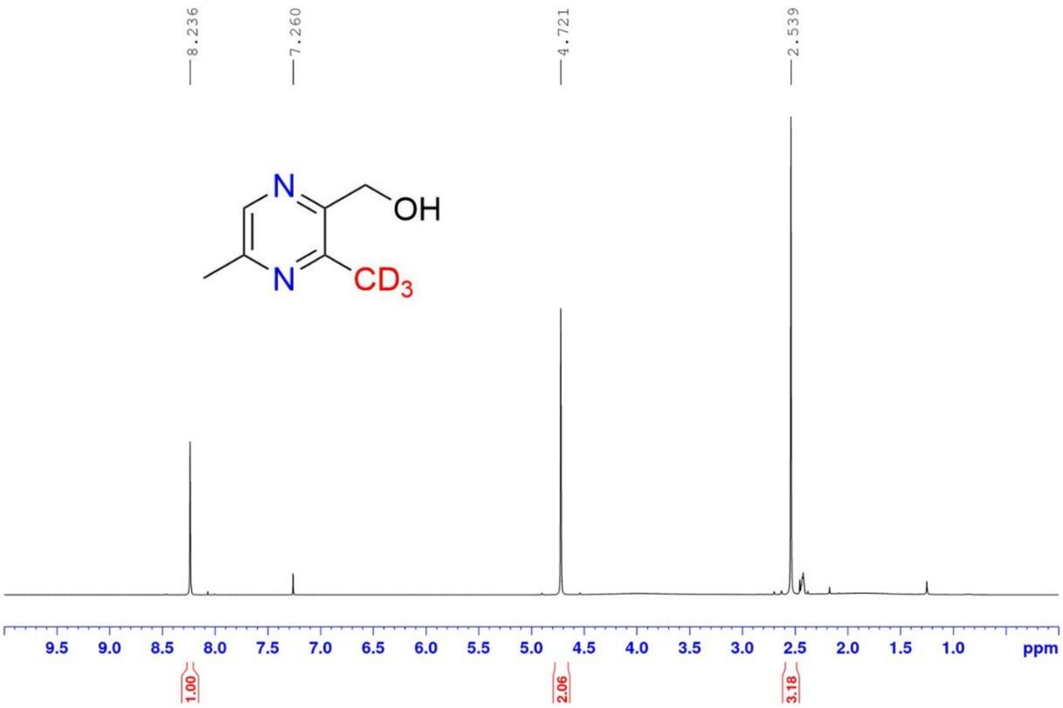

Figure S31. ^{1}H NMR (400 MHz, $CDCl_3$) of *3[$^{2}H_3$],5-Dimethyl-2-pyrazinemethanol (**2e**)*

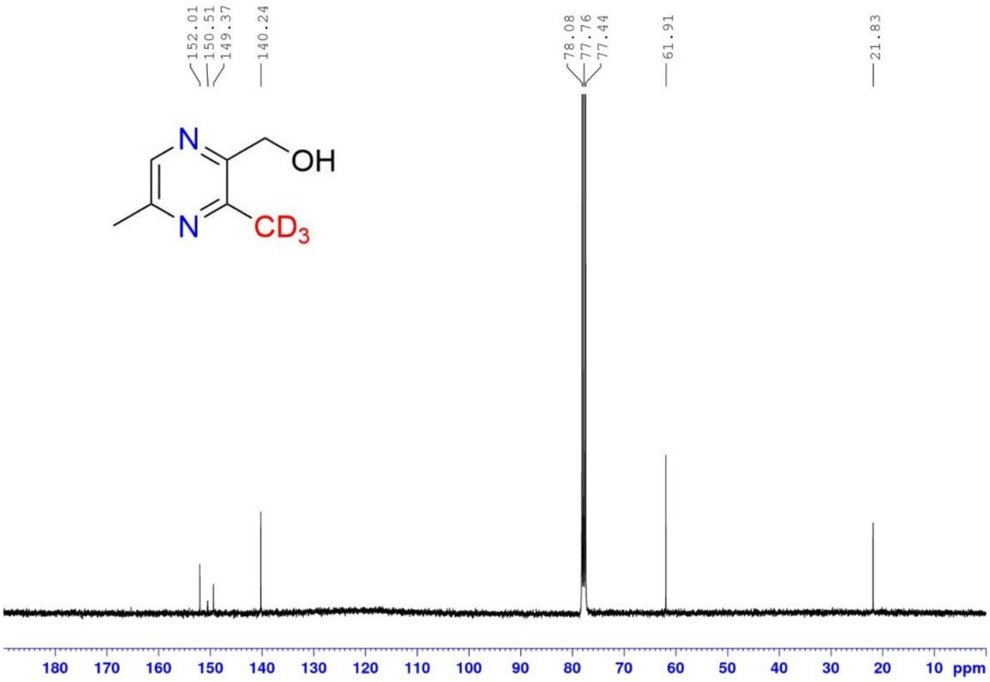

Figure S32. ^{13}C NMR (100 MHz, $CDCl_3$) of *3[$^{2}H_3$],5-Dimethyl-2-pyrazinemethanol (**2e**)*

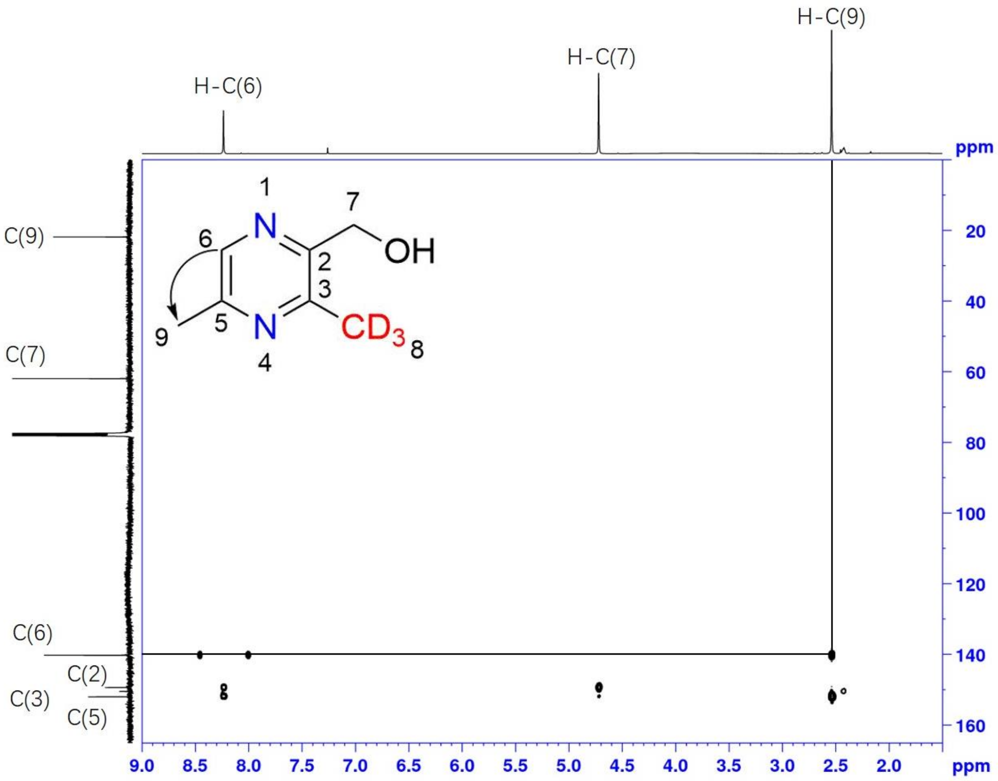

Figure S33. HMBC (400 MHz, 100 MHz, $CDCl_3$) of *3[$^2H_3$],5-Dimethyl-2-pyrazinemethanol* ***(2e)***

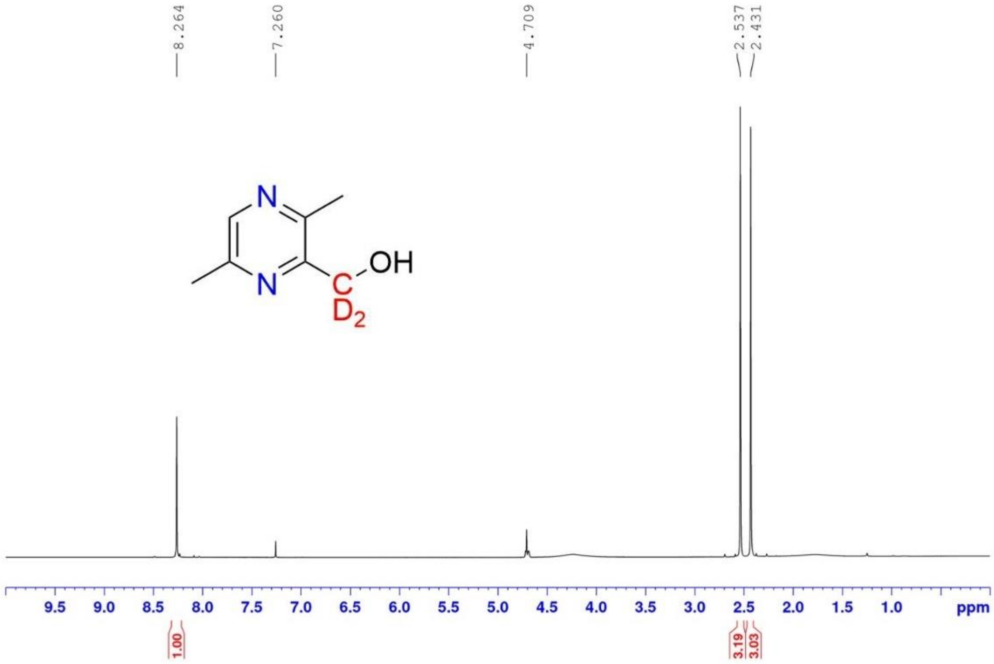

Figure S34. ^1H NMR (400 MHz, $CDCl_3$) of TMTP-d3-P2-OH

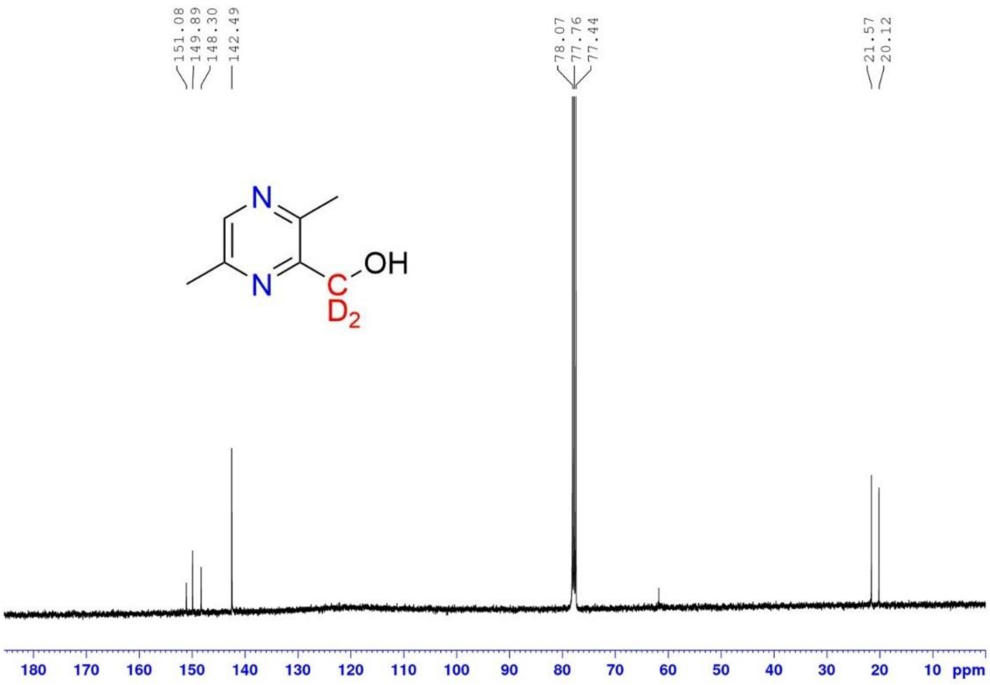

Figure S35. ^{13}C NMR (100 MHz, $CDCl_3$) of TMTP-d3-P2-OH

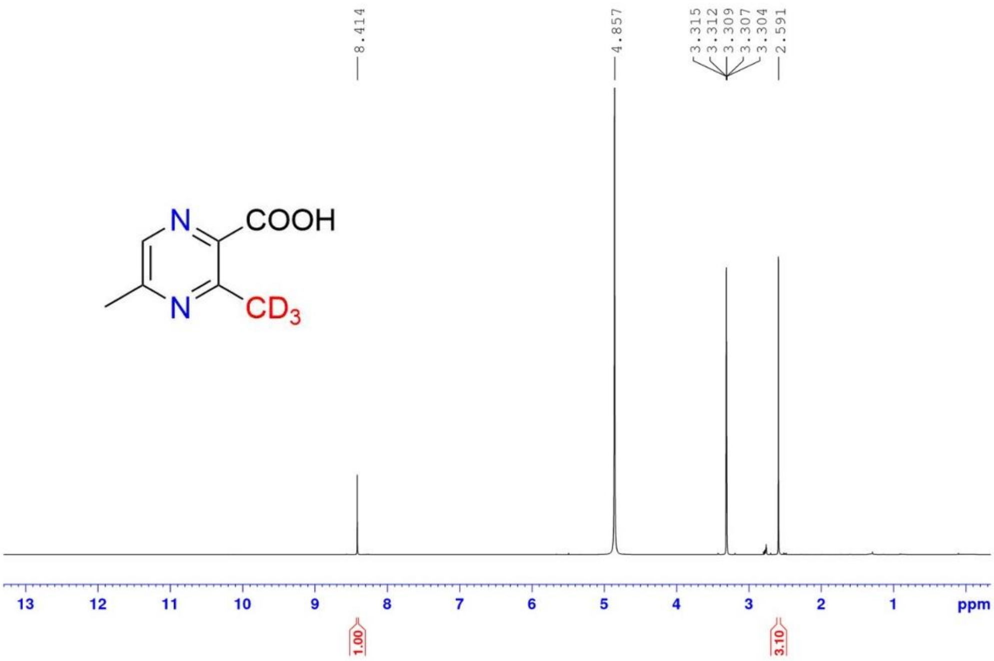

Figure S36. ^{1}NMR (600 MHz, D_2O) of *3[$^{2}H_3$],5-Dimethylpyrazine-2-carboxylic acid (**3d**)*

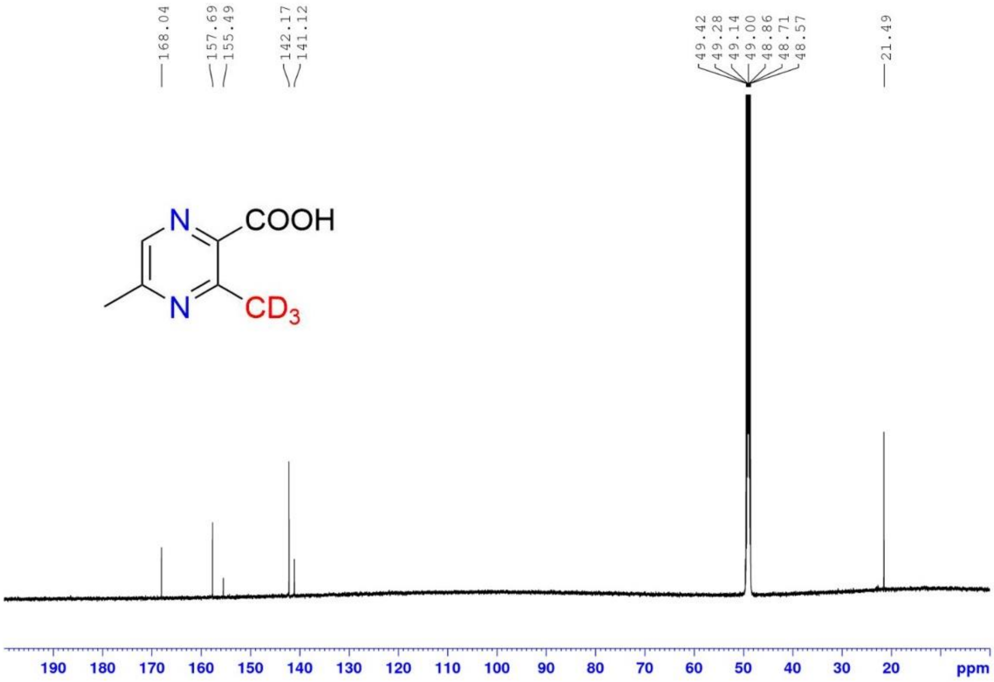

Figure S37. ^{13}C NMR (150 MHz, D_2O) of *3[$^2H_3$],5-Dimethylpyrazine-2-carboxylic acid (**3d**)*

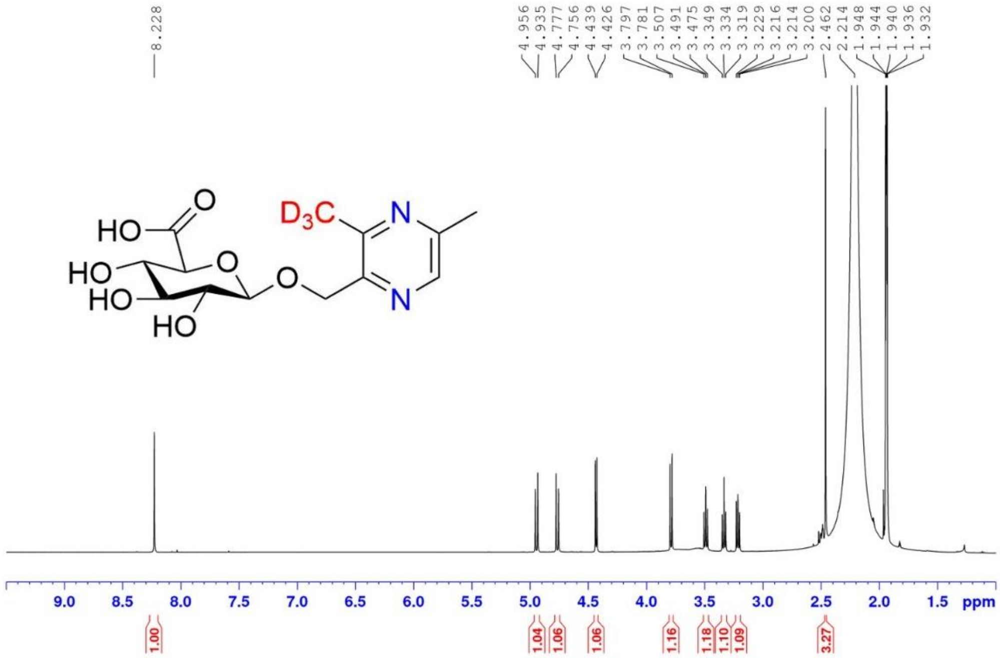

Figure S38. ^{1}H NMR (600 MHz, CD_3CN) of *(3[$^2H_3$],5-dimethylpyrazine-2-yl-)methyl-O-β-D-glucuronide (**4d**)*

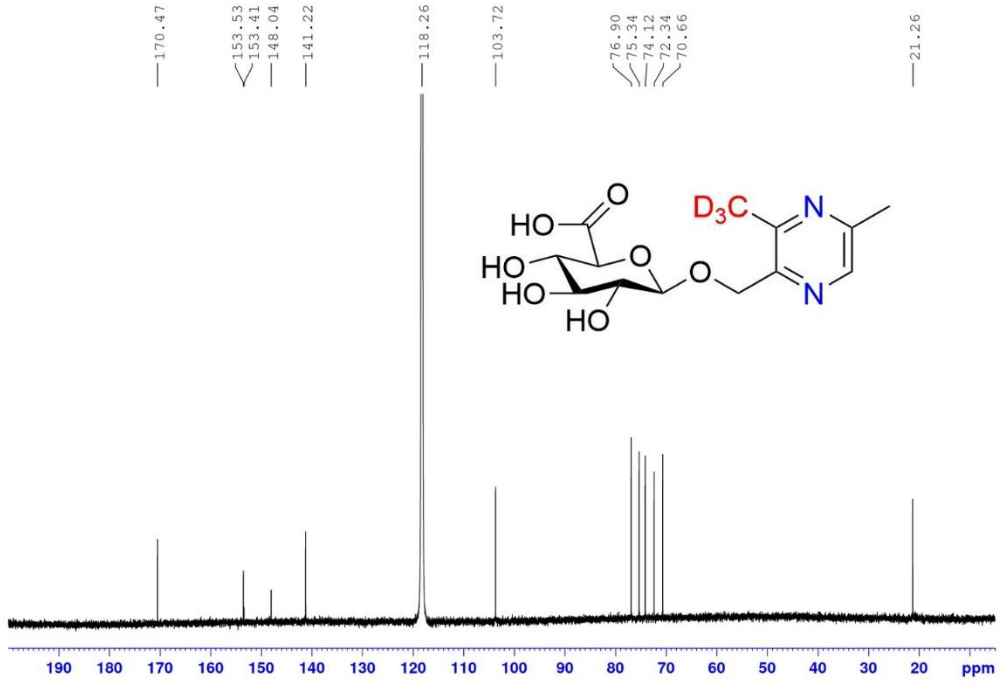

Figure S39. ^{13}C NMR (600 MHz, CD_3CN) of *(3[^{2}H$_3$],5-dimethylpyrazine-2-yl-)methyl-O-β-D-glucuronide(**4d**)*

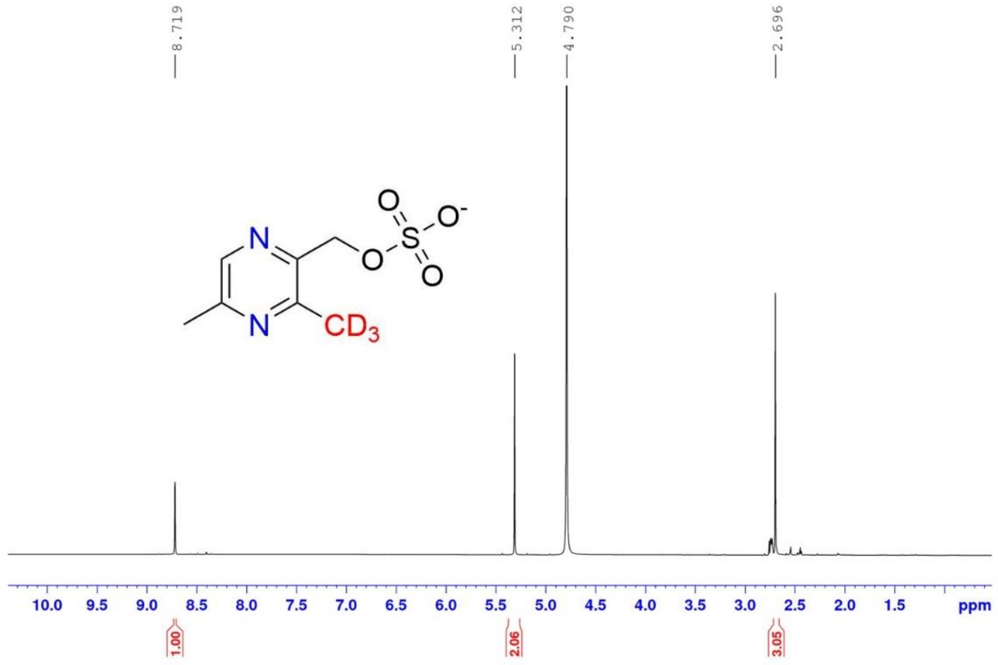

Figure S40. ^{1}H NMR (600 MHz, D_2O) of *3[^{2}H$_3$],5-dimethylpyrazine-2-yl-)methyl-sulfate (**5d**)*

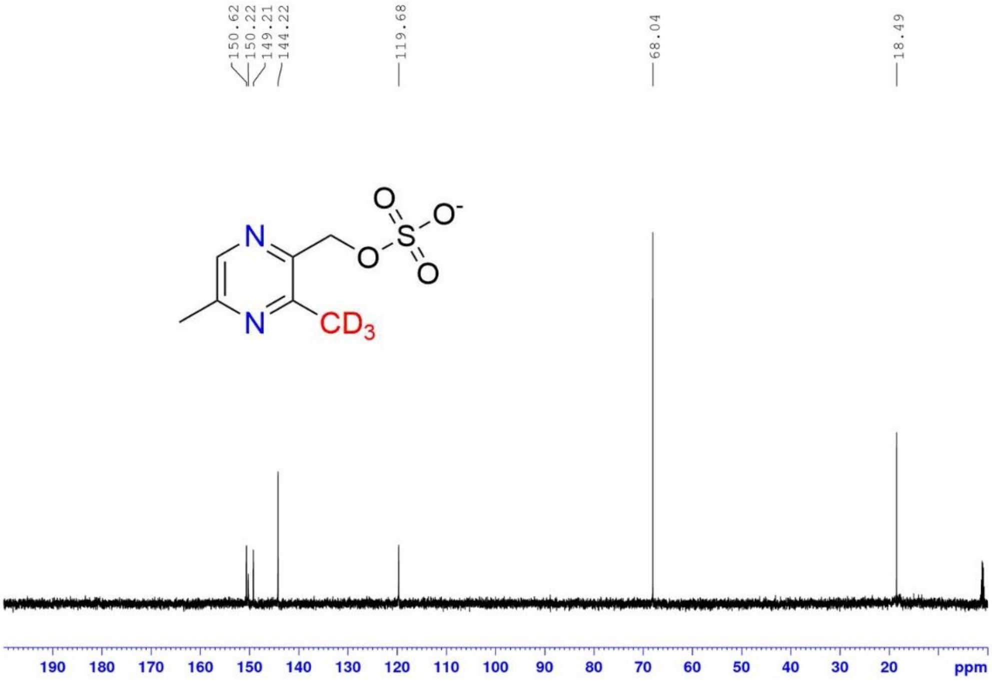

Figure S41. ^{13}C NMR (150 MHz, D_2O) of *3[$^2H_3$],5-dimethylpyrazine-2-yl-)methyl-sulfate* (***5d***)

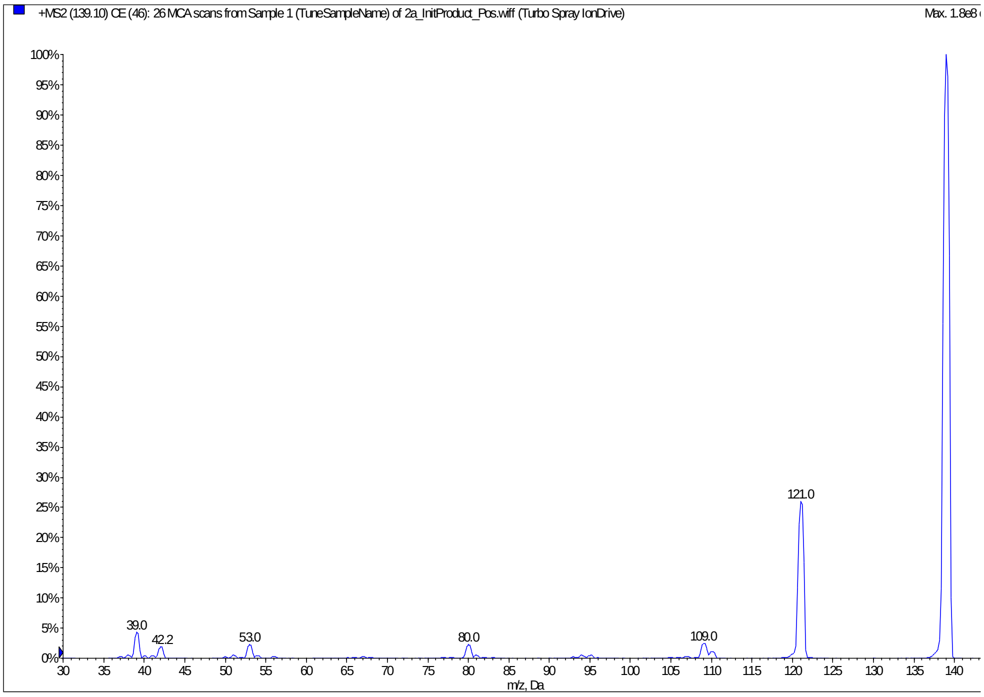

Figure S42. Product ion spectra of 3,6-dimethyl-2-pyrazinemethanol (***2a***)

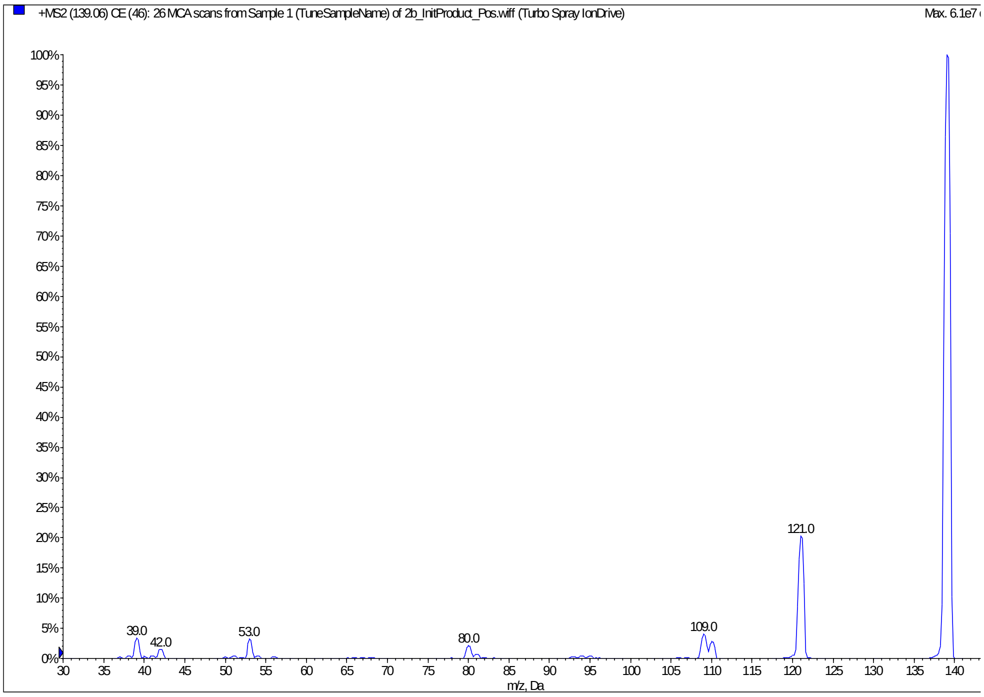

Figure S43. Product ion spectra of 3,5-dimethyl-2-pyrazinemethanol (***2b***)

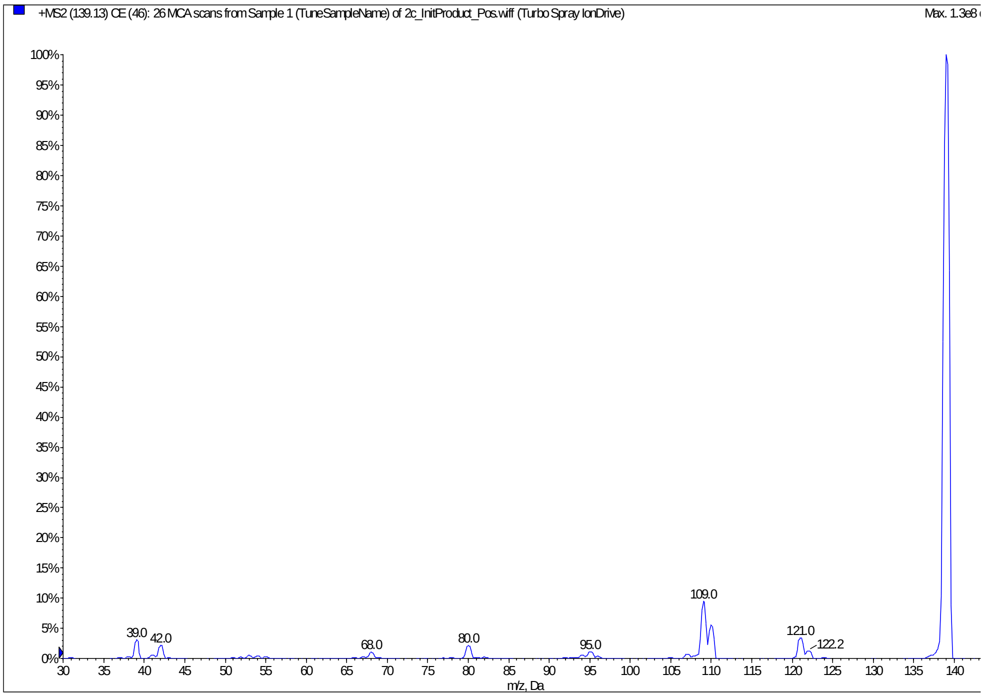

Figure S44. Product ion spectra of 5,6-dimethyl-2-pyrazinemethanol (***2c***)

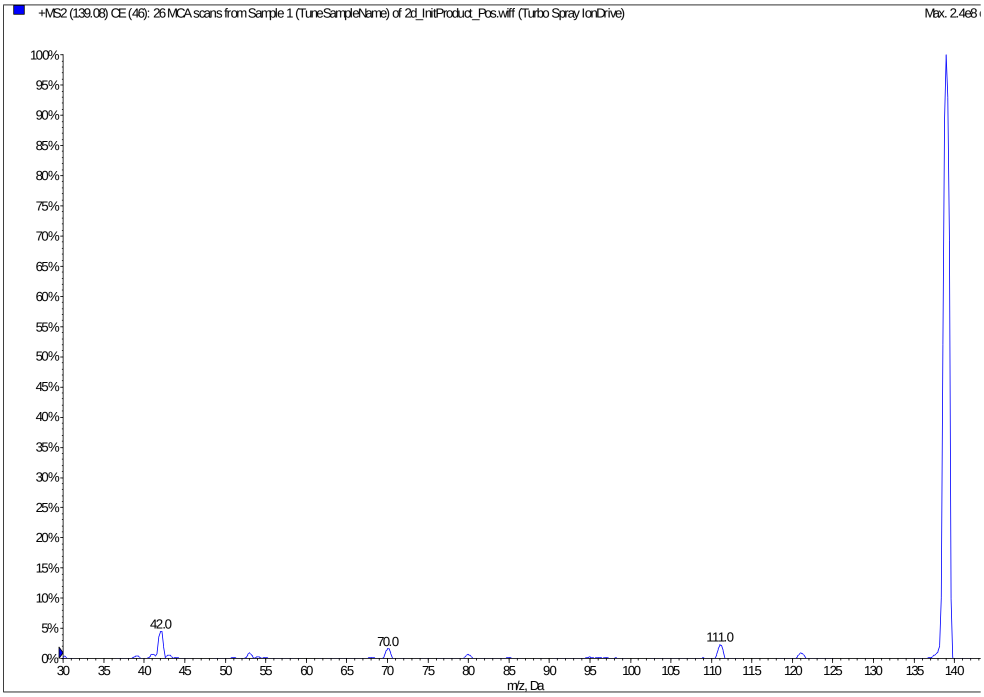

Figure S45. Product ion spectra of 3,5,6-trimethylpyrazine-2-ol (***2d***)

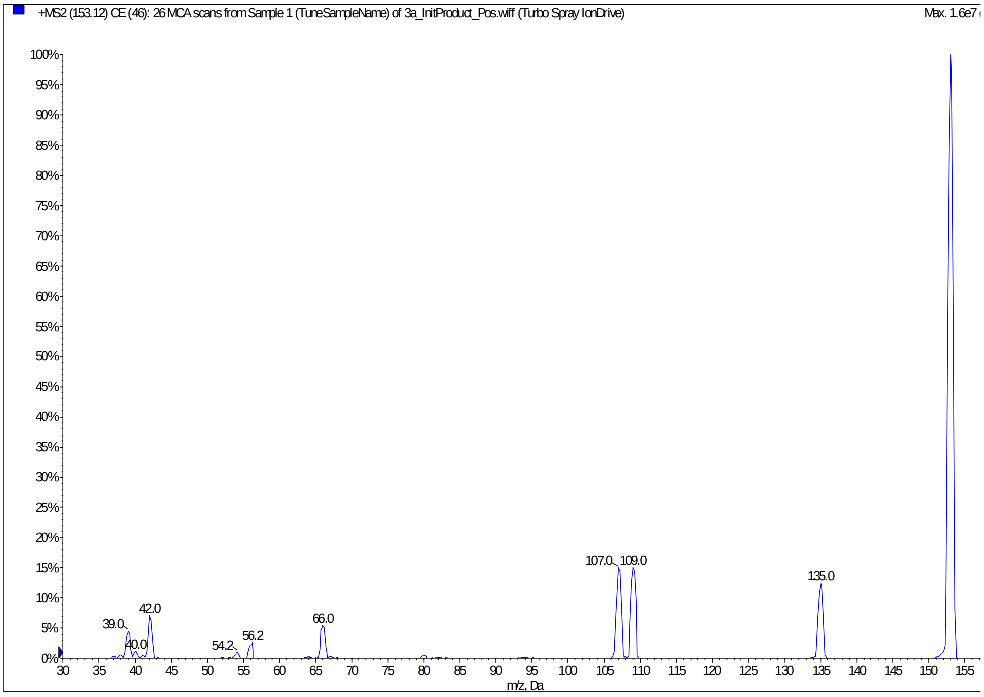

Figure S46. Product ion spectra of 3,6-dimethylpyrazine-2-carboxylic acid (***3a***)

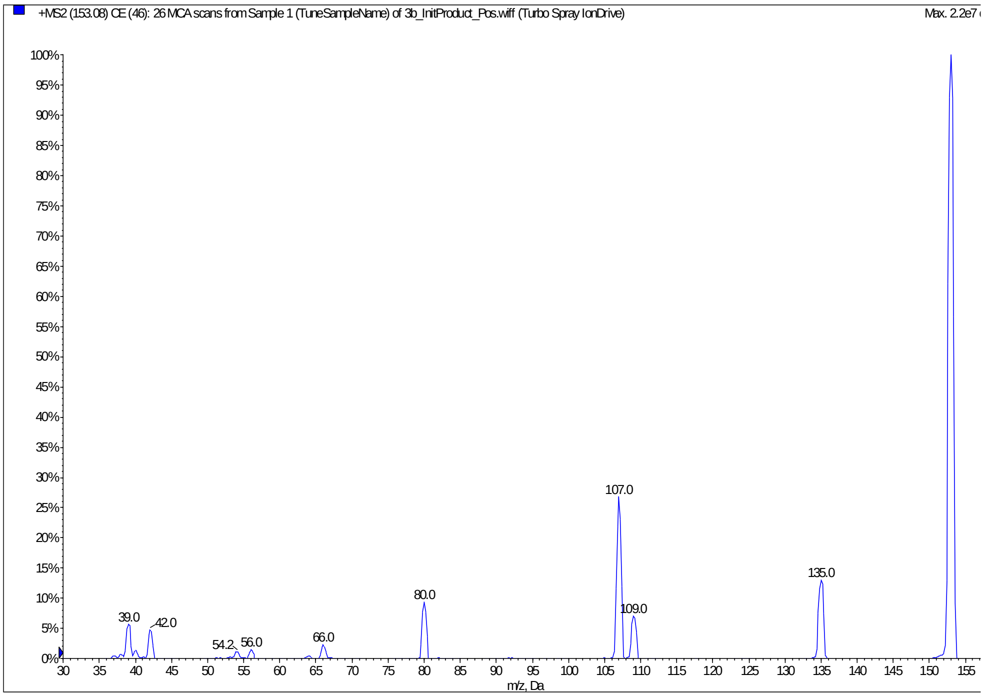

Figure S47. Product ion spectra of 3,5-dimethylpyrazine-2-carboxylic acid (***3b***)

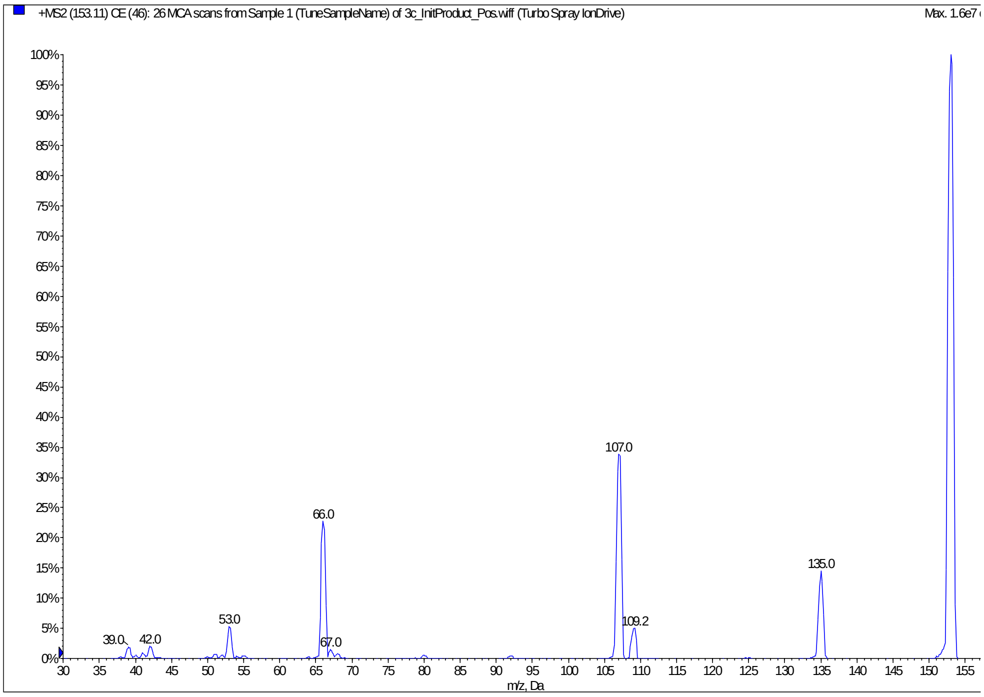

Figure S48. Product ion spectra of 5,6-dimethylpyrazine-2-carboxylic acid (***3c***)

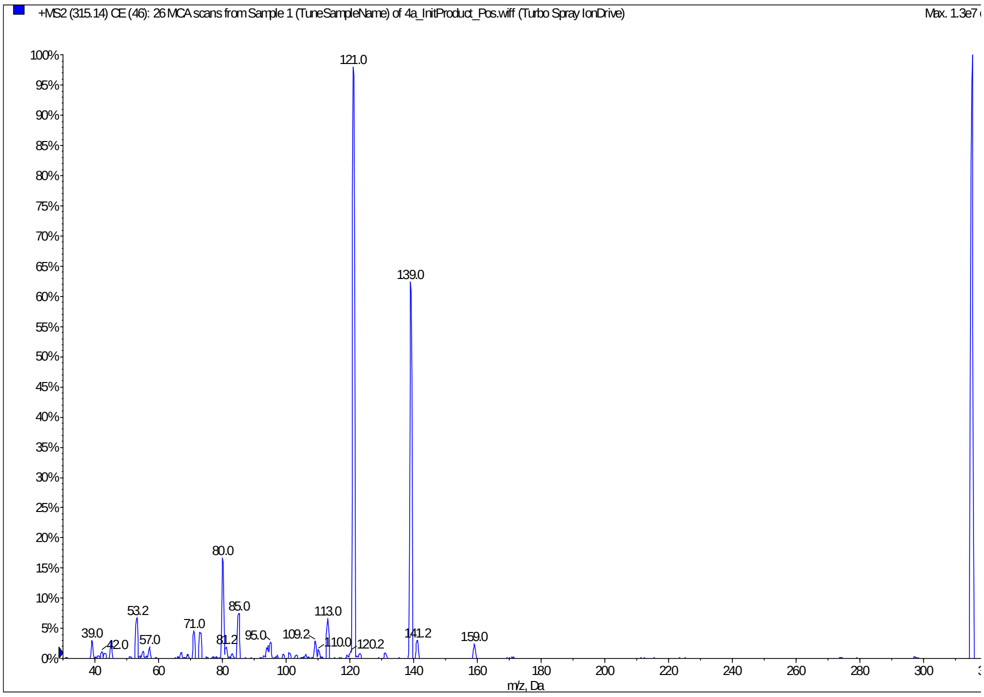

Figure S49. Product ion spectra of (3,6-dimethylpyrazine-2-yl-)methyl-O-β-D-glucuronide (***4a***)

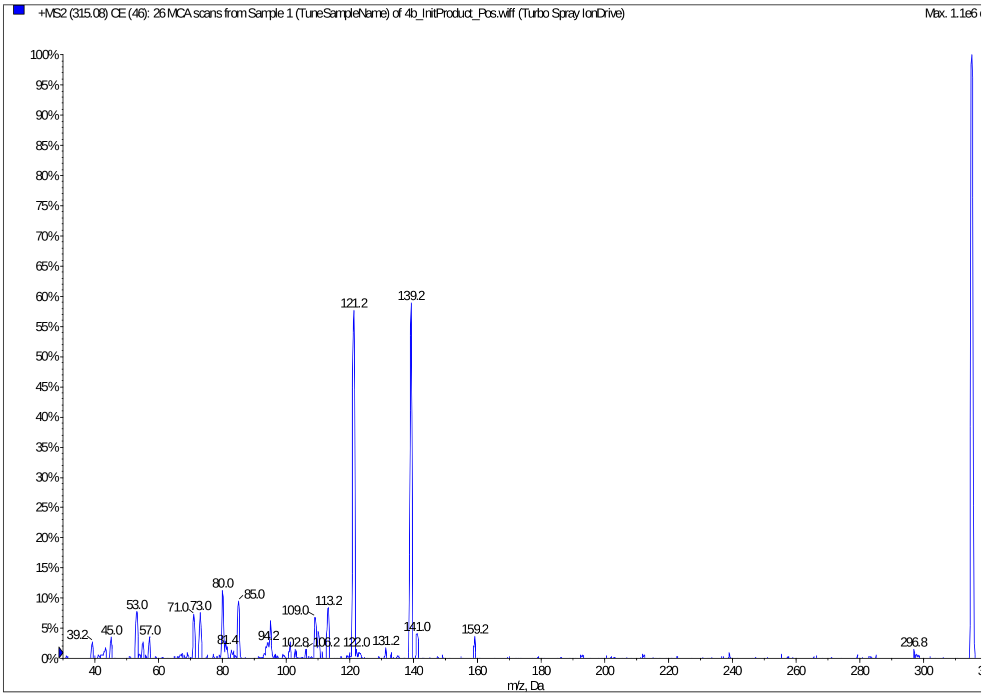

Figure S50. Product ion spectra of (3,5-dimethylpyrazine-2-yl-)methyl-O-β-D-glucuronide (***4b***)

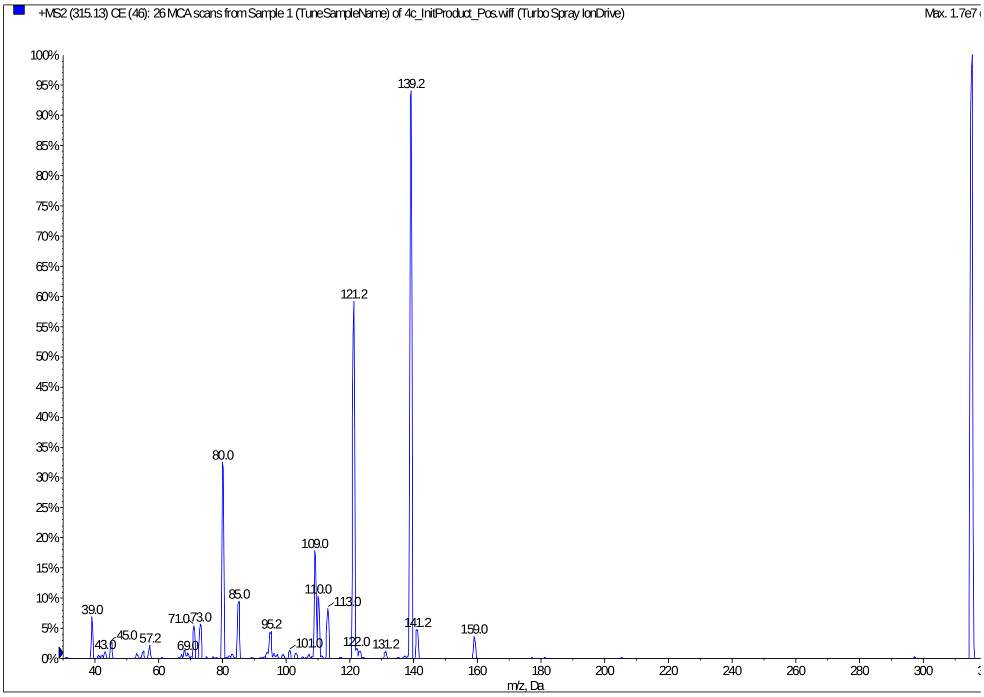

Figure S51. Product ion spectra of (5,6-dimethylpyrazine-2-yl-)methyl-O-β-D-glucuronide (***4c***)

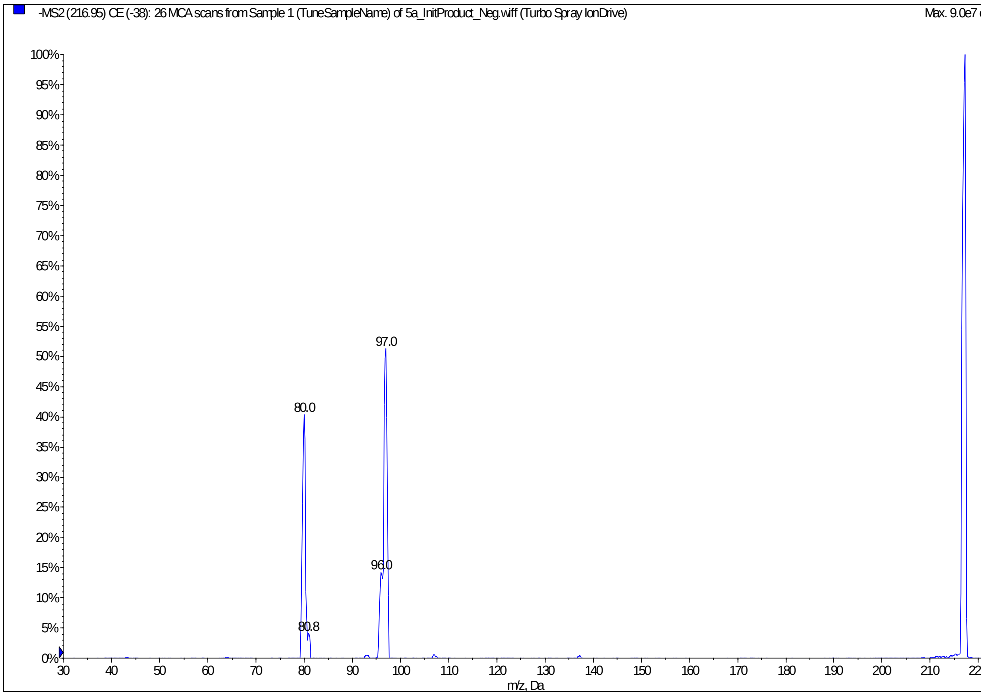

Figure S52. Product ion spectra of (3,6-dimethylpyrazine-2-yl-)methyl-sulfate (5***a***)

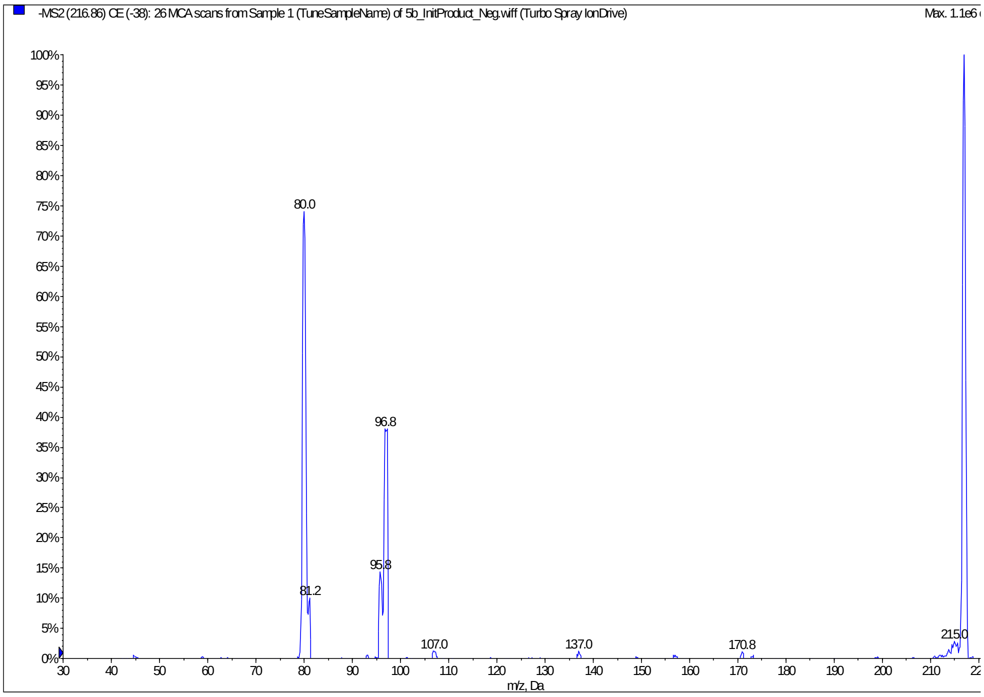

Figure S53. Product ion spectra of (3,5-dimethylpyrazine-2-yl-)methyl-sulfate (5***b***)

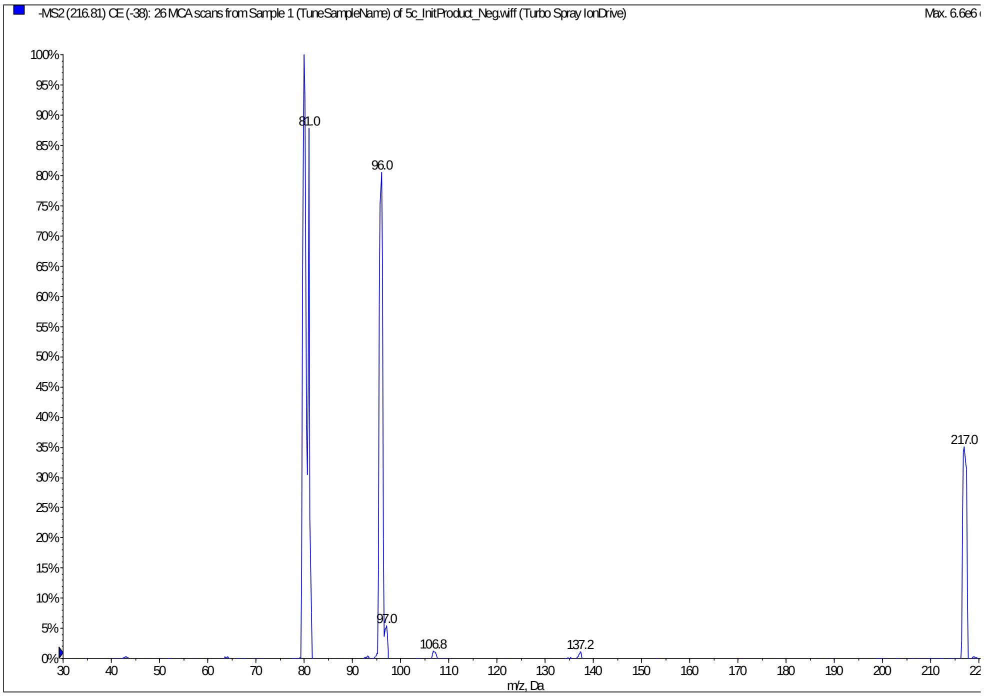

Figure S54. Product ion spectra of (5,6-dimethylpyrazine-2-yl-)methyl-sulfate (5***c***)

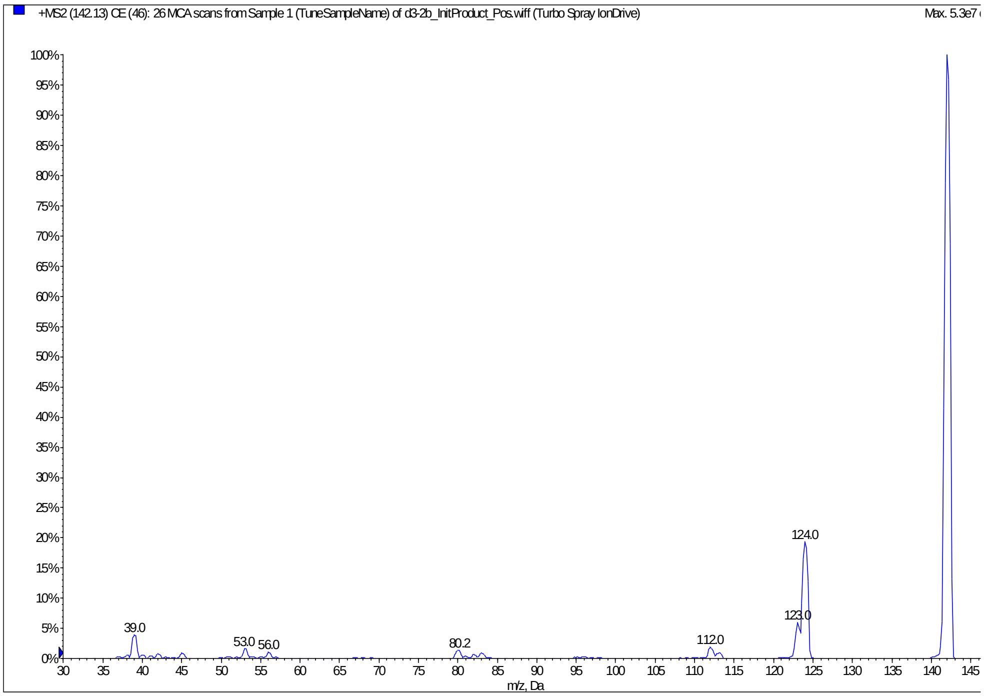

Figure S55. Product ion spectra of 3-d$_3$,5-dimethyl-2-pyrazinemethanol (***d$_3$*-2b**)

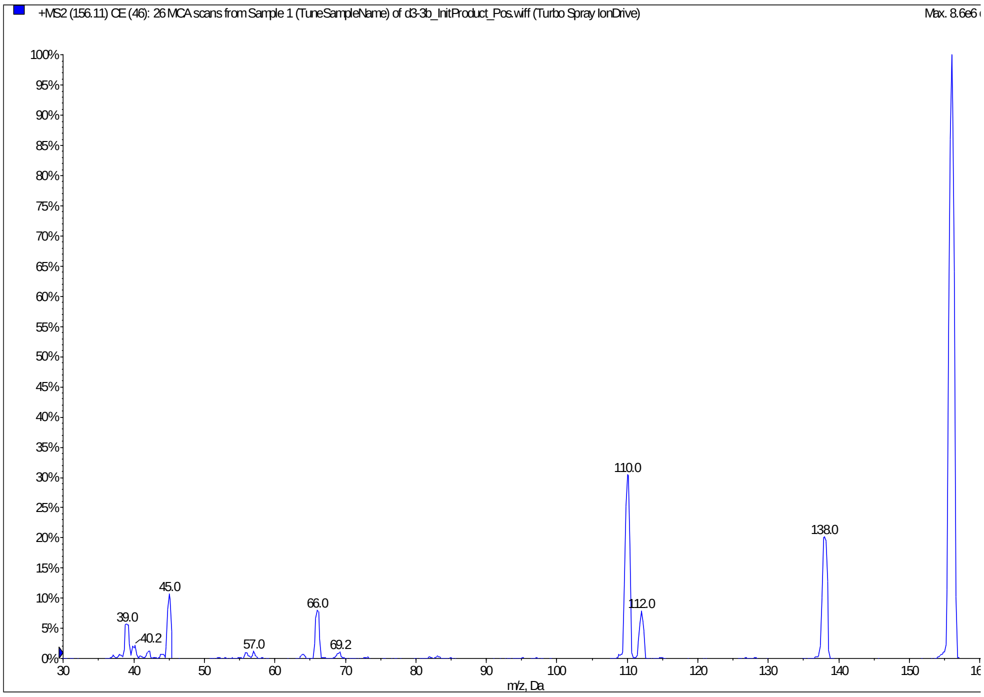

Figure S56. Product ion spectra of 3-d$_3$,5-dimethylpyrazine-2-carboxylic acid (***d$_3$*-3b**)

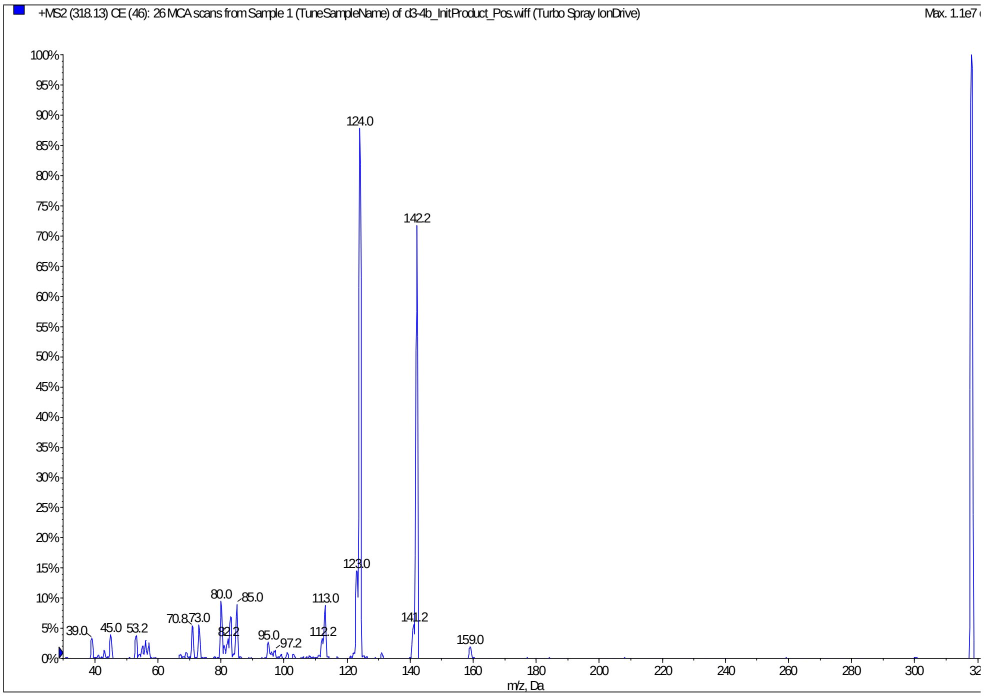

Figure S57. Product ion spectra of (3-d$_3$,5-dimethylpyrazine-2-yl-)methyl-O-β-D-glucuronide (***d$_3$*-4b**)

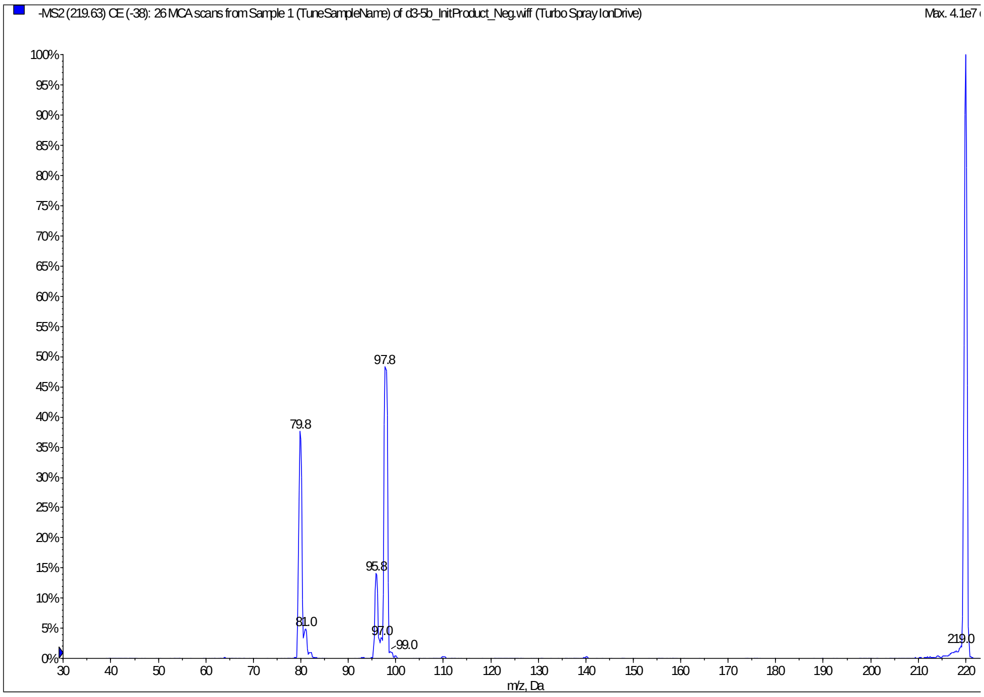

Figure S58. Product ion spectra of (3-d$_3$,5-dimethylpyrazine-2-yl-)methyl-sulfate (***d$_3$*-5b**)
